# Atomistic Understanding of 2D Monatomic Phase‐Change Material for Non‐Volatile Optical Applications

**DOI:** 10.1002/advs.202513157

**Published:** 2026-02-11

**Authors:** Hanyi Zhang, Xueqi Xing, Jiang‐Jing Wang, Chao Nie, Yuxin Du, Junying Zhang, Xueyang Shen, Wen Zhou, Matthias Wuttig, Riccardo Mazzarello, Wei Zhang

**Affiliations:** ^1^ Center for Alloy Innovation and Design (CAID) State Key Laboratory for Mechanical Behavior of Materials Xi'an Jiaotong University Xi'an 710049 China; ^2^ Institute of Physics IA RWTH Aachen University 52074 Aachen Germany; ^3^ Peter Grünberg Institute (PGI 10) Forschungszentrum Jülich GmbH 52425 Jülich Germany; ^4^ Department of Physics Sapienza University of Rome Rome 00185 Italy

**Keywords:** antimony, metavalent bonding, optical properties, phase‐change materials, thin films

## Abstract

Elemental antimony (Sb) is a promising material for phase‐change memory, neuromorphic computing, and nanophotonic applications, because its compositional simplicity prevents phase segregation upon extensive programming. Scaling down the film thickness is a necessary step to prolong the amorphous‐state lifetime. However, the optical properties of Sb are significantly altered as the thickness is reduced to a few nanometers, adding complexity to device optimization. In this work, an atomistic understanding of the thickness‐dependent optical responses is provided in Sb thin films. As thickness decreases, both the extinction coefficient and optical contrast are reduced in the near‐infrared spectrum, consistent with previous optical measurements. Such thickness dependence establishes a practical thickness limit of 2 nm, as predicted by coarse‐grained device simulations. Bonding analysis reveals a fundamentally different behavior for amorphous and crystalline Sb upon downscaling, resulting in the reduction of optical contrast. Thin film experiments are also carried out to validate our predictions. The thickness‐dependent optical trend is demonstrated by ellipsometric spectroscopy experiments, and the bottom thickness limit of 2 nm is confirmed by structural characterization experiments. Finally, it is shown that the greatly improved amorphous‐phase stability of the 2 nm Sb thin film enables robust and reversible optical switching in a silicon‐based waveguide device.

## Graphical abstract

Antimony is a promising monatomic phase‐change material. Scaling down the film thickness is necessary to prolong the amorphous‐state lifetime, but it alters the optical properties. The combined computational and experimental study shows that, as thickness decreases, the extinction coefficient and optical contrast are reduced in the near‐infrared spectrum, establishing a practical thickness limit of 2 nm for photonic applications.

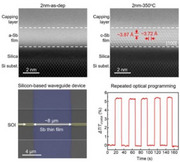

## 1 Introduction

To deal with the drastically increased demands in data storage and processing, massive research efforts have been undertaken to develop non‐volatile memory and neuromorphic computing technologies.^[^ ^1^, ^2^, ^3^, ^4^, ^5^ ^]^ Phase‐change materials (PCMs) are a leading material candidate for these applications.^[^ ^3^, ^6^, ^7^, ^8^, ^9^, ^10^, ^11^ ^]^ The basic principle is to exploit the large change in electrical and optical properties associated with the rapid and reversible phase transition between the amorphous and crystalline phases of PCMs. Conventional PCMs contain multiple elements, such as the flagship GeTe‐Sb_2_Te_3_ pseudo‐binary compounds,^[^ ^12^, ^13^, ^14^ ^]^ the typical growth‐dominant material Ag‐In‐Sb‐Te,^[^ ^15^, ^16^, ^17^ ^]^ the Sc_0.2_Sb_2_Te_3_ alloy with ultra‐high nucleation rate,^[^ ^18^, ^19^, ^20^, ^21^, ^22^ ^]^ and various new PCMs obtained by computational screening.^[^ ^23^, ^24^, ^25^ ^]^ Upon massive programming, phase segregation could occur in these multi‐component PCMs, causing device failures.^[^ ^26^, ^27^ ^]^ A promising strategy is to use only one element, e.g., Sb, for phase‐change applications.^[^ ^28^ ^]^ Although the crystallization speed of Sb can be exceedingly fast, i.e., Sb‐based electronic devices can be crystallized using sub‐nanosecond electrical pulse,^[^ ^29^ ^]^ this single‐element glass is highly unstable and crystallizes spontaneously at room temperature.^[^ ^30^ ^]^

By scaling down the film thickness to a few nanometers, the lifetime of amorphous Sb can be prolonged to tens of hours at room temperature, attributed to the confinement effects induced by the surrounding layers.^[^ ^28^, ^31^, ^32^, ^33^ ^]^ Such effects were theoretically supported by both ab initio molecular dynamics (AIMD) and machine‐learning‐driven molecular dynamics (MLMD) simulations, providing an atomistic understanding of the structural and dynamical properties of nano‐confined Sb.^[^ ^28^, ^34^ ^]^ It was also demonstrated that few‐nm amorphous Sb films can be stabilized in thin film devices and waveguide devices without any capping layer.^[^ ^35^, ^36^, ^37^ ^]^ AIMD simulations of amorphous Sb in bulk and thin film forms revealed an increasing structural dissimilarity when approaching the surface regions,^[^ ^38^ ^]^ leading to a crystallization‐suppressed slab of ≈0.7 nm per surface. This observation indicates that the bulk‐like interior could already be smaller than the critical nucleus size^[^ ^34^, ^39^ ^]^ in 2D Sb thin films. Therefore, this stabilization mechanism is an intrinsic thickness‐dependent effect, regardless of the choice of surrounding materials.

In addition to crystallization dynamics, the physical properties of Sb are also largely altered upon thickness downscaling,^[^ ^35^ ^]^ but so far, there is no theoretical report on comparing the thickness‐dependent optical properties of the amorphous and crystalline phases, which hinders further development of Sb‐based photonic devices. Take the PCM‐based waveguide device, for instance, it turns ON (with high transmission) when the PCM is switched to the amorphous phase, and turns OFF (with low transmission) upon crystallization.^[^ ^40^, ^41^, ^42^, ^43^, ^44^ ^]^ A functional all‐optical waveguide device requires both a sizable optical contrast in the telecom band between the amorphous phase and the crystalline phase and a non‐negligible extinction coefficient in both phases to enable sufficient Joule heating for phase transition. However, there should exist a bottom thickness limit for Sb, because as the film thickness is reduced toward the 2D limit, crystalline Sb undergoes a major change in electronic structure from metallic to semiconducting,^[^ ^45^ ^]^ leading to a high transmission state^[^ ^46^ ^]^ in the monolayer and few‐layer Sb.^[^ ^47^ ^]^ Besides, the optical changes in the visible light range can also be exploited for non‐volatile color display applications.^[^ ^35^, ^48^ ^]^

In this work, we perform systematic density functional theory (DFT) calculations to understand the thickness dependence of electronic structure and optical profiles in both crystalline (c‐) and amorphous (a‐) Sb films from sub‐nm up to ≈5.1 nm, enabling a direct comparison with the spectroscopic ellipsometry measurements. In combination with the coarse‐grained finite‐element method (FEM) modeling, our multiscale simulations predict a bottom limit of ≈2 nm with a sufficient programming window for a functional Sb waveguide device. We provide an explanation on the trends of optical variations from a chemical bonding perspective. Moreover, we carry out structural and optical experiments on Sb thin films and confirm the bottom thickness limit of Sb thin films to be ≈2 nm, which corresponds to only 12 atomic layers in the crystalline phase, as evidenced by cross‐sectional scanning transmission electron microscopy (STEM) experiments. Our electrical and optical measurements prove that this 2D Sb thin film still has sizable contrast in physical properties upon crystallization, which are sufficiently large to enable practical phase‐change applications.

## 2 Results and Discussion

**Figure 1:**
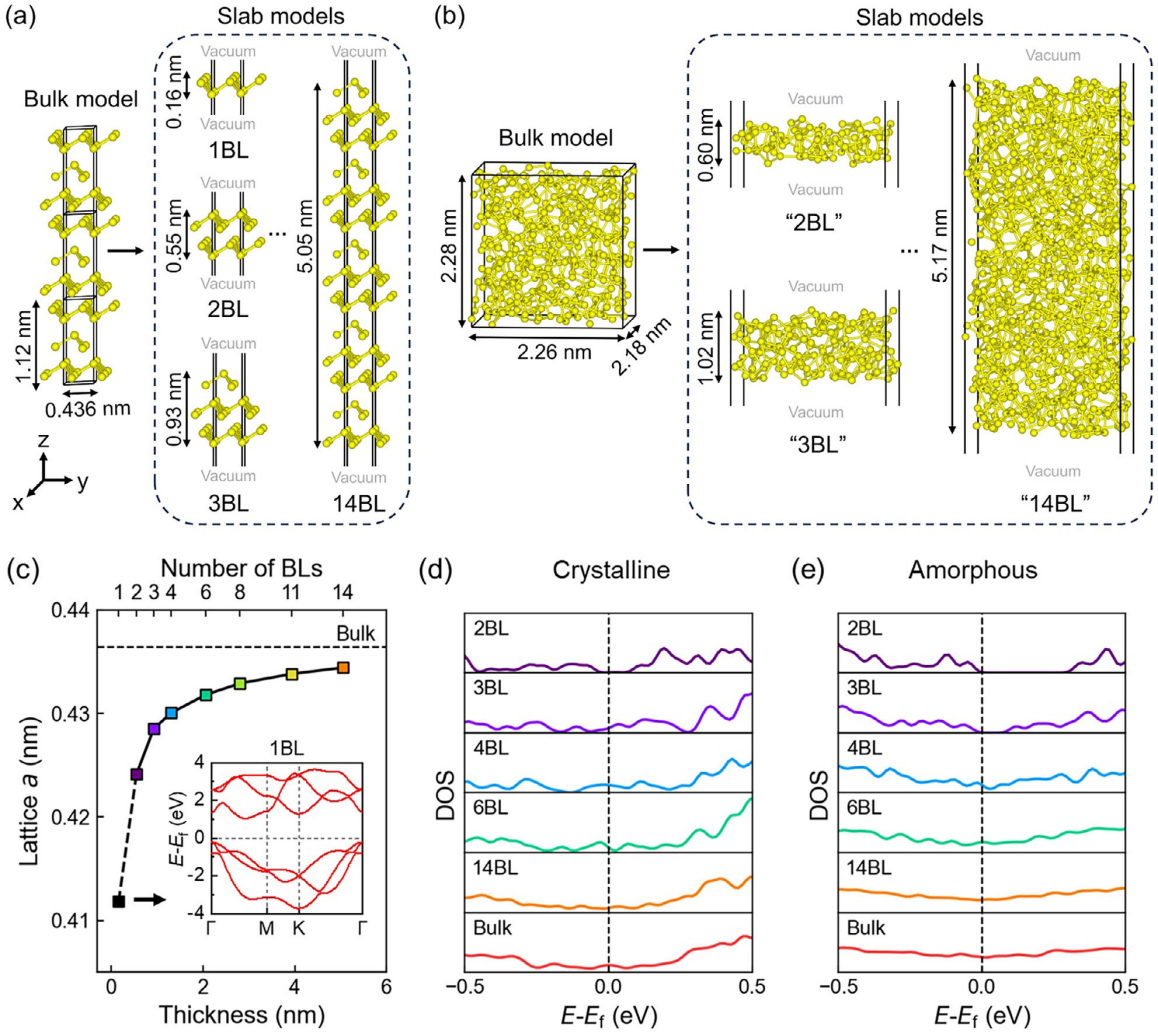
DFT modeling of bulk and slab models of Sb. a) The relaxed crystalline bulk and slab models. All crystalline models were built using hexagonal unit cells, except the *z*‐axis. b) The calculated amorphous bulk and slab models. All amorphous models were built using orthorhombic cells. c) The optimized in‐plane lattice parameter of crystalline models as a function of thickness. The inset shows the band structure of the 1 BL model. The DOS profiles calculated for d) crystalline and e) amorphous models.

It is straightforward to build slab models for c‐Sb by adding a vacuum slab of 2.5 nm along the *z*‐axis. We considered a set of models ranging from 1 bilayer (BL) antimonene up to 14 BL slab models (**Figure** **1**a) with a film thickness of ≈0.2–5.1 nm. It is noted that in the case of 1 BL, the structure can be viewed as a buckled monolayer. Hence, antimonene is also termed as a monolayer (ML) in the literature. For 2 BL and thicker models, there is a non‐negligible covalent interaction between the BLs due to the long Sb─Sb bonds of ≈3.35 Å, similarly to the case of GeTe slabs.^[^ ^49^ ^]^ In the following, we denote the building blocks as “BL” instead of “ML”. As regards the amorphous phase, three melt‐quenched a‐Sb bulk models with independent thermal histories were first generated using AIMD.^[^ ^38^ ^]^ Each model contained 360 atoms in a box of 2.26 × 2.18 × 2.28 nm^3^. Next, we constructed a‐Sb slab models according to the thickness of c‐Sb models. For instance, we simulated cutting a slab of ≈0.6 nm (size of the 2BL c‐Sb model) out of a bulk a‐Sb model, and annealed such a model in the presence of a 2.5 nm vacuum slab at 300 K over 30 ps. We denoted this a‐Sb slab model as “2 BL” as well (Figure 1b). This a‐Sb model of ≈0.6 nm basically represents the bottom limit in thickness. The “1 BL” a‐Sb model cannot be generated properly, because Sb atoms tend to form clusters laterally (Figure S1, Supporting Information). For thicker models (>2.28 nm), we used two and even three bulk a‐Sb models to build the slab. Two or three sub‐slabs taken from different bulk models were glued together, and the interfaces between different sub‐slabs were properly relaxed to avoid too short chemical bonds or big voids. The largest a‐Sb slab model we constructed was “14 BL” (≈5.1 nm), containing 798 atoms (Figure 1b). For each thickness, two additional a‐Sb slab models were generated for statistics. For thick models (>2.28 nm), additional models were generated by integrating the sub‐slabs from different parts of the three‐bulk a‐Sb models. The lattice variation of c‐Sb models upon downscaling is shown in Figure 1c, showing that the in‐plane lattice parameter *a* was gradually reduced from 0.436 nm (bulk) to 0.412 nm. This reduction in lattice parameter in ultrathin c‐Sb films is consistent with the observation of the blueshift in Raman spectroscopy experiments.^[^ ^35^ ^]^ This lattice contraction phenomenon was also reported for other c‐PCMs.^[^ ^49^, ^50^, ^51^ ^]^

Prior to the electronic structure and optical calculations, all the bulk and slab models were further relaxed at 0 K. The semiconducting nature of the 1 BL model is reproduced with our DFT calculation, and the calculated band structure is shown in Figure 1c inset (gap size ≈1.26 eV). For thicker models, we calculated their density of states (DOS) to enable a direct comparison between the crystalline phase (Figure 1d) and the amorphous phase (Figure 1e). A sizable energy gap can still be observed in the crystalline model of 2 BL and the amorphous models of up to 4 BL (see the data for two other amorphous configurations in Figure S2, Supporting Information), but for thicker models, the gap is filled.

Next, we carried out calculations of the real (*ε*_1_) and imaginary (*ε*_2_) parts of the dielectric function for these bulk and slab models. The frequency‐dependent dielectric functions were calculated within the independent‐particle approximation, which was shown to be adequate to characterize the optical properties of PCMs.^[^ ^52^, ^53^, ^54^, ^55^, ^56^ ^]^ The refractive index (*n*) and extinction coefficient (*k*) were calculated using the following formulas:^[^ ^57^ ^]^

(1) $$n={\left(\frac{\sqrt{\varepsilon_{1}^{2}+\varepsilon_{2}^{2}}+\varepsilon_{1}}{2}\right)}^{\frac{1}{2}}\;$$

(2) $$k={\left(\frac{\sqrt{\varepsilon_{1}^{2}+\varepsilon_{2}^{2}}-\varepsilon_{1}}{2}\right)}^{\frac{1}{2}}$$

These frequency‐dependent optical data can be used to make a direct comparison with experimental results.

**Figure 2:**
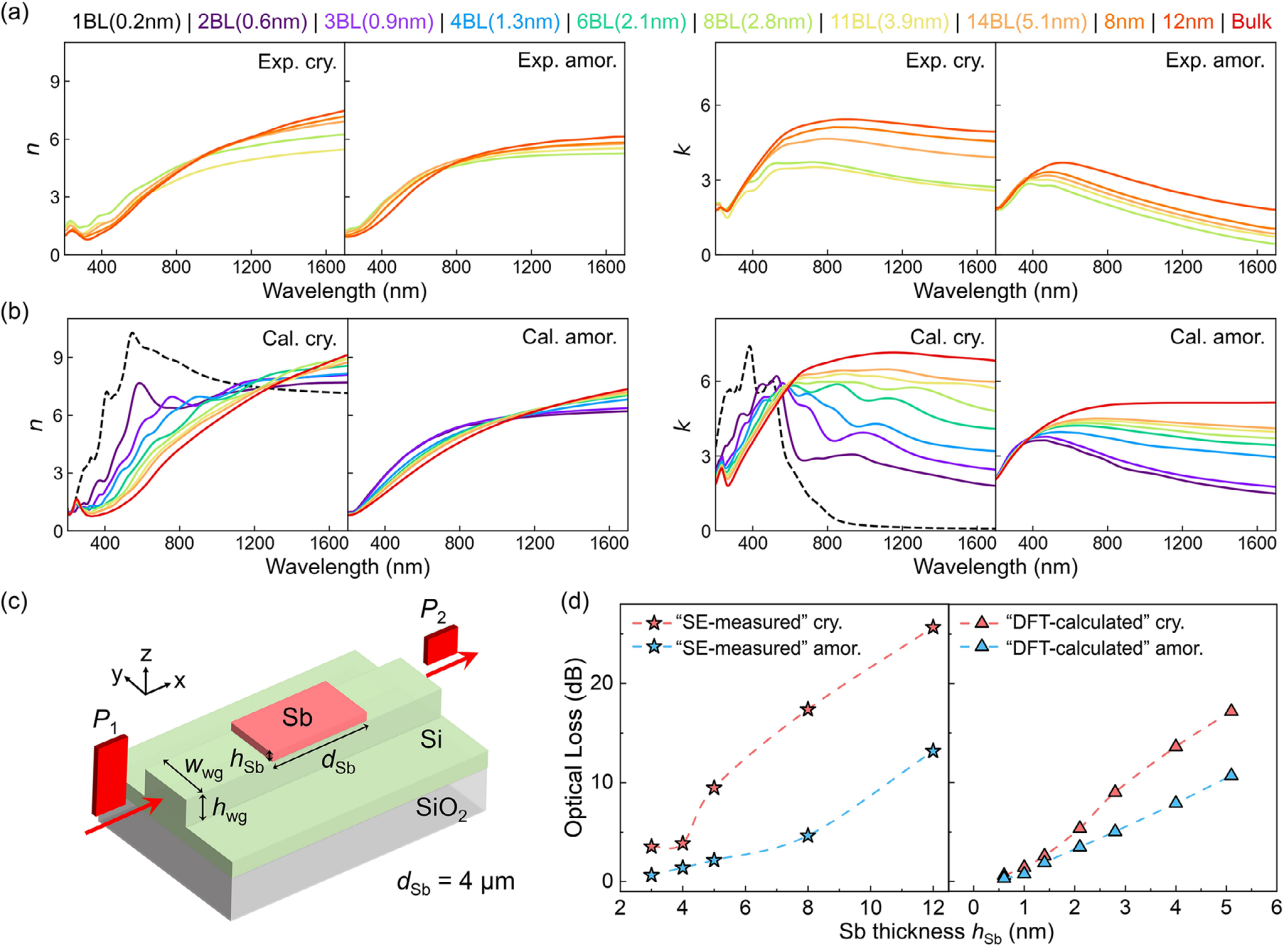
Comparison between optical properties of Sb from experiments and DFT calculations. Refractive index (*n*) and extinction coefficient (*k*) in the spectrum range between 200 and 1700 nm for crystalline (cry.) and amorphous (amor.) antimony thin films with varying thicknesses. a) The experimental (Exp.) data obtained by spectroscopic ellipsometry measurements, taken from Ref.[35] with permission. b) The computational results (Cal.) have been obtained in this work. c) A schematic of the antimony‐based photonic waveguide studied in our FEM simulations. d) The optical loss of waveguides at the telecommunication C‐band (1550 nm) from FEM simulations. The optical data of Sb films from DFT calculations (“DFT‐calculated”, this work) and spectroscopic ellipsometry measurements (“SE‐measured”, Ref.[35]) were used to describe the optical response of antimony.

The optical profiles of the as‐deposited and post‐annealed Sb thin films in the absence of capping layers were measured via spectroscopic ellipsometry experiments in Ref.[35] According to their Raman spectroscopy measurements, no oxidation issue was found for the Sb thin films. Here, we replot the optical data measured for the Sb thin films of ≈3–12 nm in **Figure** **2**a. As the thickness decreases, the refractive index *n* of crystalline films shows an increase in the visible‐light range, but a reduction in the near infrared range. A crossover is observed at ≈1000 nm. A similar thickness‐dependent trend in *n* is observed in the amorphous films, but with smaller variations and an earlier onset of crossover at ≈800 nm. As regards the extinction coefficient *k*, both crystalline and amorphous Sb films show a clear reduction as the thickness decreases, covering the visible‐light and near infrared ranges. Also, a larger contrast window between the thinnest and thickest films is observed in the crystalline films than in the amorphous ones.

These optical features are overall consistent with our DFT calculations, as shown in Figure 2b. The dependence of *n* with thickness in both crystalline and amorphous films shows opposite trends in two different wavelength regions, with crossover points at ≈1300 and ≈1050 nm, respectively. The same holds for the experimental data (Figure 2a), although the crossover points occur at smaller wavelengths. As for *k*, the crystalline and amorphous films exhibit overall reduction with decreasing thickness. In addition, the thickness dependence of these properties is less pronounced in amorphous films than in crystalline films, implying a diminution of the optical contrast with the decrease in thickness. The data for the other two sets of amorphous models is consistent with Figure 2b and are shown in Figure S3 (Supporting Information). It should be noted that these DFT calculations were carried out with the standard PBE functional, which is less accurate in describing the size of the energy gap. Therefore, it is not feasible to reproduce exactly the same optical values as those obtained experimentally. In addition, our DFT calculations are based on simplified models that cannot fully capture the polycrystalline nature of the experimental samples. Nevertheless, the qualitative trend in optical properties as a function of film thickness is well reproduced by our DFT calculations. In short, both experiments and DFT calculations consistently find a reduced optical contrast between a‐Sb and c‐Sb in the infrared spectrum as the film thickness decreases.

Moreover, our DFT calculations provide additional information to supplement experimental results. Our slab models cover the thickness range from 1 bilayer to ≈5 nm, greatly extending the lower limit of experimental data (i.e., 3 nm). As shown in Figure 2b, the optical properties of ultra‐thin films (<2 nm) are notably different from thicker films. In the visible‐light region, both the refractive index *n* and the extinction coefficient *k* of crystalline ultra‐thin films are visibly higher than for thicker films. In the near‐infrared, a much lower *k* is expected in either phase for ultra‐thin films.

With the thickness‐dependent optical data, we were able to perform a series of FEM simulations using COMSOL (see Methods) to investigate the contrast window of Sb‐based photonic memory devices to approach the thickness limit. We focused on a standard silicon‐on‐insulator waveguide memory device (sketched in Figure 2c) with the height and width of the waveguide *h*_wg_ and *w*_wg,_ being set as 0.12 and 0.45 µm, consistent with Ref.[36] Regarding the parameters of the Sb film, we considered a normal material length *d*_Sb_ = 4 µm, and varied the film thickness *h*_Sb_ from 0.6 nm up to 12 nm. Based on the input and output power of waveguides (*P*_1_ and *P*_2_, respectively), we can calculate their transmittance (*T* = *P*_2_/*P*_1_) and optical loss (−10lg(*P*_2_/*P*_1_)).

The calculated optical loss data as a function of *h*_Sb_ are presented in Figure 2d. For a given thickness *h*_Sb_, we used both the measured and calculated *n* and *k* of thin film samples or models when available. We referred to these two sets of FEM simulations as “spectroscopic ellipsometry (SE)‐measured” and “DFT‐calculated” in the following. A more pronounced reduction in optical loss was observed for the crystalline state than the amorphous state with the decrease of *h*_Sb_, reducing the contrast window. This trend holds for both SE‐measured and DFT‐calculated data. For the amorphous phase, the change in optical loss follows a nearly linear trend as *h*_Sb_ varies, while a sharper change was observed for the crystalline state in the range between ≈5 and ≈3 nm in the SE‐measured curve and between ≈3 nm (8 BL) and ≈1.3 nm (4 BL) in the DFT‐calculated curve. Notably, the contrast between the two phases became no longer visible at 1.3 nm (4 BL) and below.

**Figure 3:**
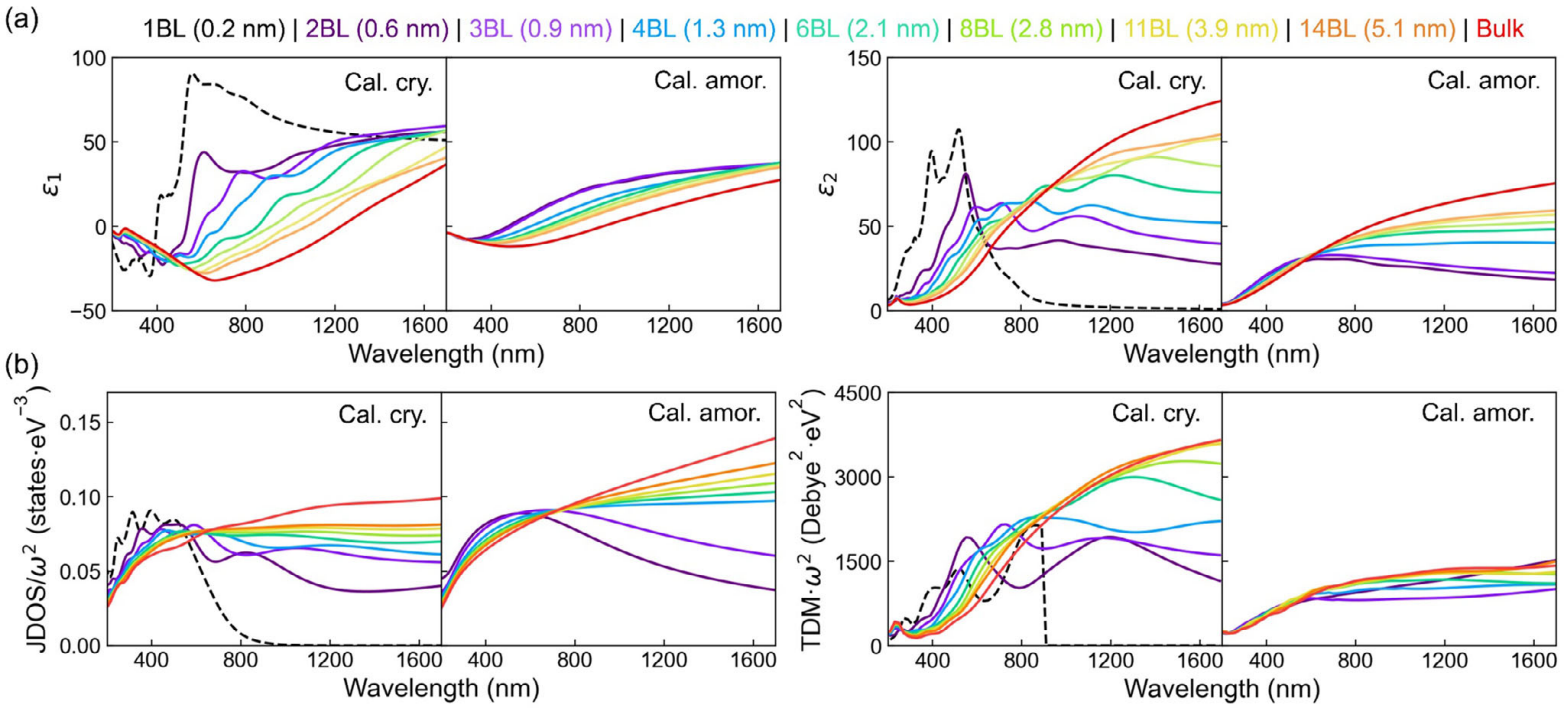
The real (*ε*_1_) and imaginary (*ε*_2_) parts of the dielectric function and the two contributing factors to *ε*_2_. a) The real (*ε*_1_) and imaginary (*ε*_2_) parts of the frequency‐dependent dielectric function for crystalline and amorphous antimony models. b) Joint density of states (JDOS) and transition dipole moment (TDM). *ω* refers to the photon energy. The original TDM data contains a massive number of scatter points, with each point showing the transition probability of one possible excitation. Here, the TDM points at each wavelength were averaged to make a line plot, for clarity.

We examine the underlying mechanism for such thickness dependence of the optical properties. As revealed by Equations (1) and (2), the change in refractive index is a direct result of the evolution of the dielectric functions. **Figure** **3**a presents the calculated real (*ε*_1_) and imaginary (*ε*_2_) parts of the dielectric function. With the reduction of thickness, *ε*_1_ increases in both crystalline and amorphous films. For *ε*_2_, there is a notable decrease in the infrared region for both states, and an additional increase below ≈600 nm for the crystalline films. Importantly, crystalline films show more evident variations with thickness than amorphous films. To further explain such a trend, the two contributing factors to *ε*_2_, namely the joint density of states (JDOS) and the transition dipole moment (TDM),^[^ ^52^, ^53^ ^]^ were calculated and presented in Figure 3b. They quantify the amount of inter‐band excitations and transition probability, respectively, with the former one reflecting the effects of the electronic DOS and the latter one characterizing the degree of electron delocalization. For JDOS, both crystalline and amorphous phases show notable reduction with decreasing thickness in the near‐infrared region, as a consequence of the opening of an energy gap and the resulting reduction in the DOS near the Fermi level. However, for TDM, crystalline films exhibit a dramatic reduction, while amorphous films show a much smaller variation. This observation implies that the decrease of film thickness results in the localization of electrons in the crystalline phase but has a modest impact on the amorphous phase, which accounts for the more obvious change in the optical properties in the crystal.

The evolution in the degree of electron delocalization can be further understood in terms of bonding mechanisms. It has been reported that amorphous PCMs exhibit ordinary covalent bonding, while crystalline PCMs are stabilized by metavalent bonding^[^ ^58^, ^59^, ^60^, ^61^, ^62^, ^63^, ^64^, ^65^, ^66^ ^]^ (MVB). Distinct from ordinary covalent bonding based on electron pairing (i.e., two electrons per bond), MVB is characterized by extensive *p* orbital alignment and approximately one *p* electron per bond on average, giving rise to electron delocalization. As a result, metavalently bonded solids show a large chemical bond polarizability, leading to high values of the Born effective charge as well as a large optical dielectric constant.^[^ ^59^ ^]^ But the *p* orbital alignment is broken in the amorphous phase, and the electrons become more localized, leading to a much weaker optical response. This is the reason why PCMs have a large optical contrast between the two states.^[^ ^67^ ^]^ In previous works, two bonding indicators have been employed to distinguish MVB from other bonding types, namely the number of electrons transferred (ET) and electrons shared (ES) between pairs of neighboring atoms.^[^ ^61^ ^]^ These quantities can be obtained from DFT calculations. MVB features intermediate ES values, i.e., smaller than those of ordinary covalent bonding but larger than metallic bonding values, as well as moderately small ET.^[^ ^61^ ^]^

**Figure 4:**
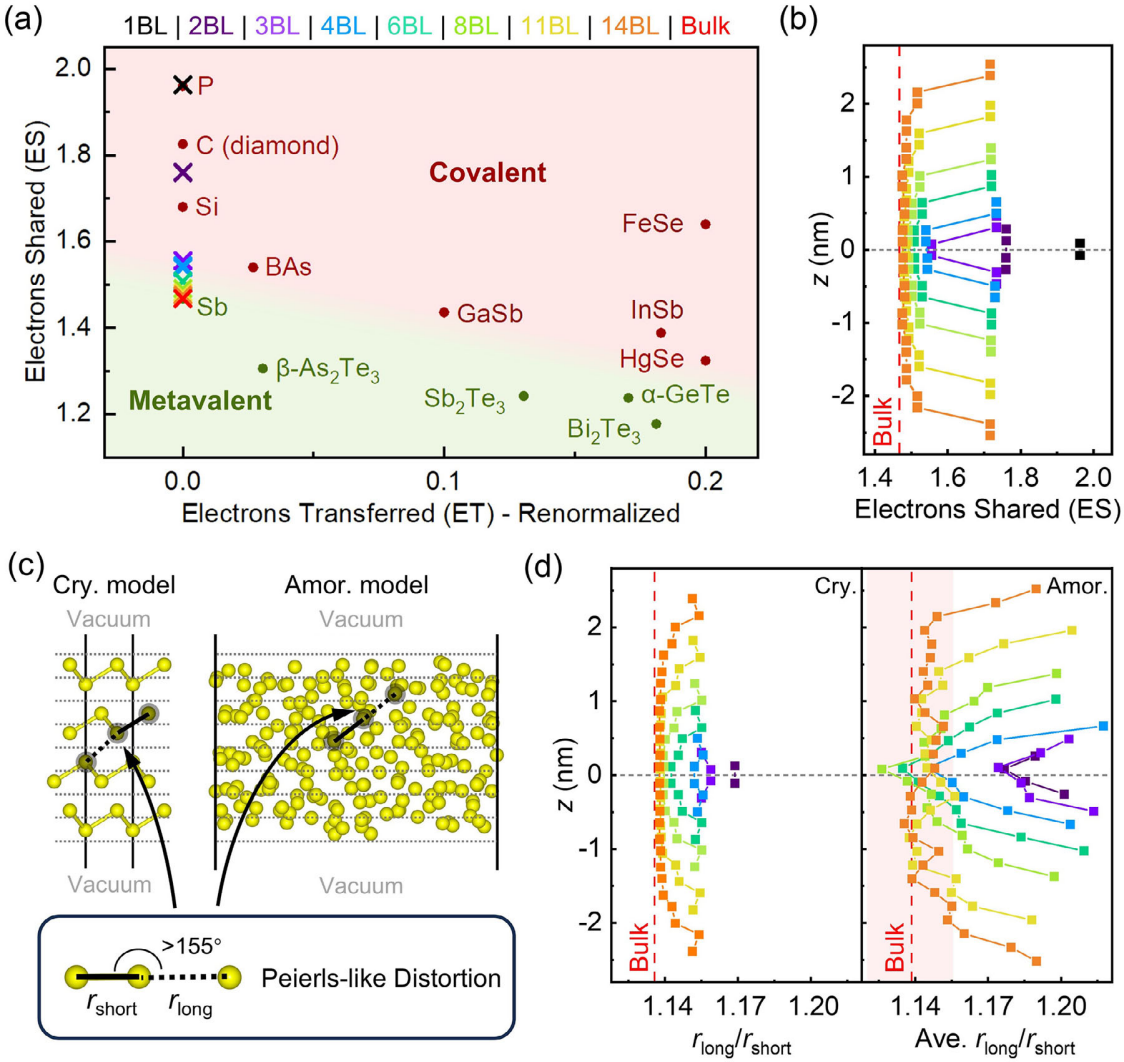
Chemical bonding analysis for crystalline and amorphous models. a) The locations of the crystalline models on the bonding map defined by the Electrons Transferred (ET) and Electrons Shared (ES) indicators. The crosses represent the antimony models in this work, while dots stand for data for other materials adapted with permission from Ref.[63] b) The ES for each atomic layer in crystalline slab models. *z* refers to the coordinates of the layers along the *z*‐axis. c) A sketch illustrating the definition of Peierls distortion (PD). The grey dotted lines indicate the separation of each slab model into layers for PD analysis. d) PD (*r*_long_/*r*_short_) for each layer in crystalline (Cry.) and amorphous (Amor.) models. The red shaded area shows the fluctuation range of PD in bulk amorphous models.

To analyze the evolution of the bonding character as a function of film thickness, we calculated the ET and ES values of the c‐Sb models (see Methods), and presented them on an ET‐ES bonding map (**Figure** **4**a). Since there is only one element in all the Sb models, their ET values are always zero. The ES values refer to the shortest bond of the particular atom. The ES value of a typical covalently bonded monatomic crystal (e.g., black phosphorus) is ≈2, and that of a typical metallic crystal (e.g., silver) is ≈0.5.^[^ ^67^ ^]^ The ES value of Sb atoms in the bulk rhombohedral model is thus calculated to be 1.47. For slab models, we calculated the ES values of each atom along the *z*‐axis, and the distributions are shown in Figure 4b. For the 1BL model, the ES value is almost 2, indicating a highly localized bonding nature. As the thickness is doubled, the ES values of all four Sb atoms reduce sharply to 1.76. For thicker slab models, the bonding character of the atoms in the center shows a major difference as compared to the outer bilayers. The ES value of the former is gradually reduced from 1.56 to 1.48, but that of the atoms at the edge remains high, ≈1.72. The ES value of the central atom of each slab model is plotted in Figure 4a. As the thickness increases, the ES value changes from a position comparable to black phosphorus to that of bulk Sb, indicating a change in bonding character from covalent‐like to metavalent‐like. The 6BL (≈2 nm) model seems to be a “critical” point, which also shows a large increase in TDM (Figure 3b). These findings indicate that electron delocalization requires relatively long‐range and collective orbital interaction and alignment.

Peierls distortion (PD), namely, the ratio of long and short bonds (*r*_long_/*r*_short_) in a well‐aligned bonding pair (Figure 4c), is another important indicator in characterizing the bonding characteristics,^[^ ^65^, ^68^, ^69^ ^]^ which can be easily extended to highly disordered solids. We performed a layer‐by‐layer analysis of *r*_long_/*r*_short_ for both c‐Sb and a‐Sb models (Figure 4d). The PD profiles of the c‐Sb slab models provide insights consistent with the calculated ES values. Few‐layer c‐Sb slab models below 3BL show much more severe PD than the bulk c‐Sb model. For thicker c‐Sb slab models, the PD in the interior part always shows a lower value than that in the surface bilayers (the long Sb─Sb bonds are slightly increased by ≈0.04 Å at the surfaces due to the less compact bonding environment), and is weakened further as an increase of thickness. We divided the amorphous slab models into layers according to the thickness of a single layer in bulk c‐Sb (≈0.2 nm), and calculated the average *r*_long_/*r*_short_ for each layer (Figure 4d, right panel). Similar to the crystalline models, the surface layers (≈0.7 nm thick) show larger PD than the center region. However, contrary to the crystalline models, where the weakening of PD with the increasing thickness is evident until 11BL (≈4 nm), the amorphous films show bulk‐like PD in the center as early as 4BL (≈1.3 nm). Due to the disordered atomic structure of a‐Sb, electron orbitals are misaligned, leading to localized orbital interactions. Therefore, the bonding properties of a‐Sb are much less affected by the thickness, and the evolution in TDM is thus modest.

According to the analyses of PD (Figure 4d) and coordination number (Figure S4, Supporting Information), the “4BL” and even thinner amorphous models show high structural dissimilarity as compared to the bulk amorphous phase. This feature indicates that nucleation could be difficult at or below such a thickness of ≈1.3 nm. Besides, a major change of bonding character from covalent‐like to metavalent‐like occurs in the range of 4–6 BL (1.3–2.1 nm) in the crystalline state, accompanied by an evident increase in excitation probability and hence a notable rise in optical contrast. Therefore, this range should be regarded as the bottom limit for a functional Sb thin film with both crystallization capacity and sizable contrast window.

**Figure 5:**
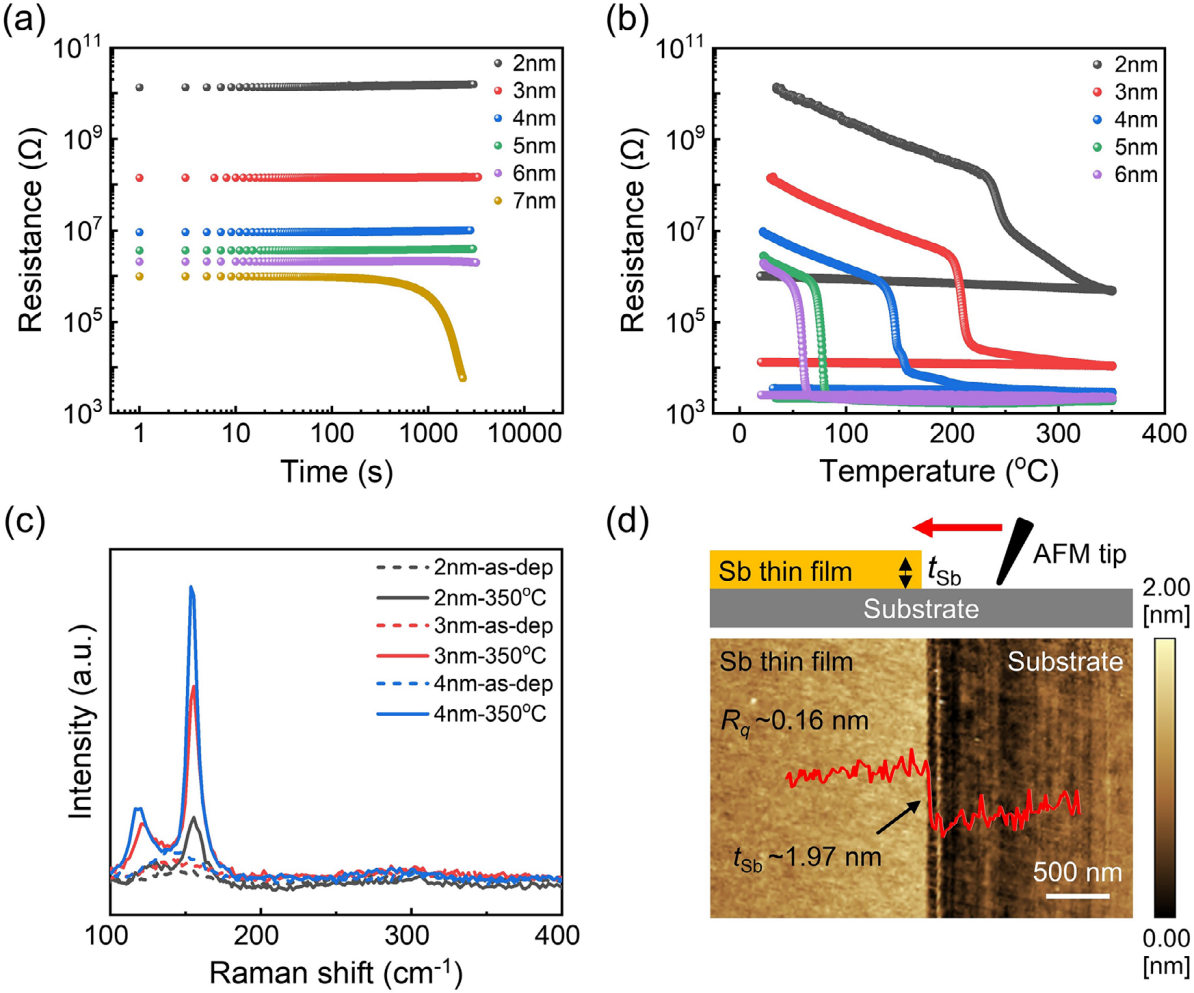
Preparation and characterizations of Sb thin films. a) The changes in sheet resistance of Sb thin films at room temperature over time. b) The measured sheet resistance of Sb thin films as a function of temperature. c) The Raman spectra of as‐deposited and thermally annealed Sb thin films. d) The atomic force microscopy measurements of ≈2 nm as‐deposited a‐Sb thin film.

To validate the theoretical prediction, we prepared Sb thin films via magnetron sputtering on silicon substrates with thicknesses of 1–7 nm. The thin films were all covered with a standard non‐conductive capping layer of ≈10 nm ZnS:SiO_2_ for the electrical measurements as a function of time or temperature. As shown in **Figure** **5**a, the 7 nm a‐Sb thin film started to crystallize after 100 s, but other thinner films can sustain the amorphous form over a longer time. Upon in situ heating to 350 °C with a heating rate of 10 °C min^−1^, the 6 and 5 nm a‐Sb thin films got crystallized quickly at *T*_x_ of 58 and 77 °C, showing relatively poor thermal stability (Figure 5b). Further downscaling largely enhanced the *T*_x_ to be 145, 209 and 243 °C for the 4, 3, and 2 nm case, respectively. Note that the *T*_x_ of a flagship PCM Ge_2_Sb_2_Te_5_ is ≈150 °C. Our Raman spectra (Figure 5c) confirmed that after heating, these thin films indeed crystallized, as seen by the emergence of *E*_g_ (at ≈120 cm^−1^) and *A*_1g_ peaks (at ≈155 cm^−1^). No oxidation was observed in our samples, as the peak corresponding to Sb_2_O_3_ was absent in the Raman spectra. For even thinner films below 2 nm, however, the electrical resistance became too high, exceeding the measurable range, and there was no clear signature of crystallization from the Raman spectra.

To assess the thickness variation and uniformity, we carried out an atomic force microscopy (AFM) experiment on an as‐deposited a‐Sb thin film of 2 nm with no capping layer. As shown in Figure 5d, the overall thickness of the thin film was measured as ≈1.97 ± 0.16 nm, confirming an atomically flat surface morphology. However, direct thermal annealing to 350 °C would result in a cluster issue, and the deposition of a capping layer is necessary to obtain 2 nm crystalline Sb thin films of high smoothness. Remarkably, this 2 nm Sb thin film still exhibited a resistance window of over four orders of magnitude (Figure 5b), and the amorphous stability was largely enhanced with a high *T*_x_ of 243 °C. These features make 2 nm Sb a superior option for electronic nonvolatile memory applications as compared to thicker Sb films.

**Figure 6:**
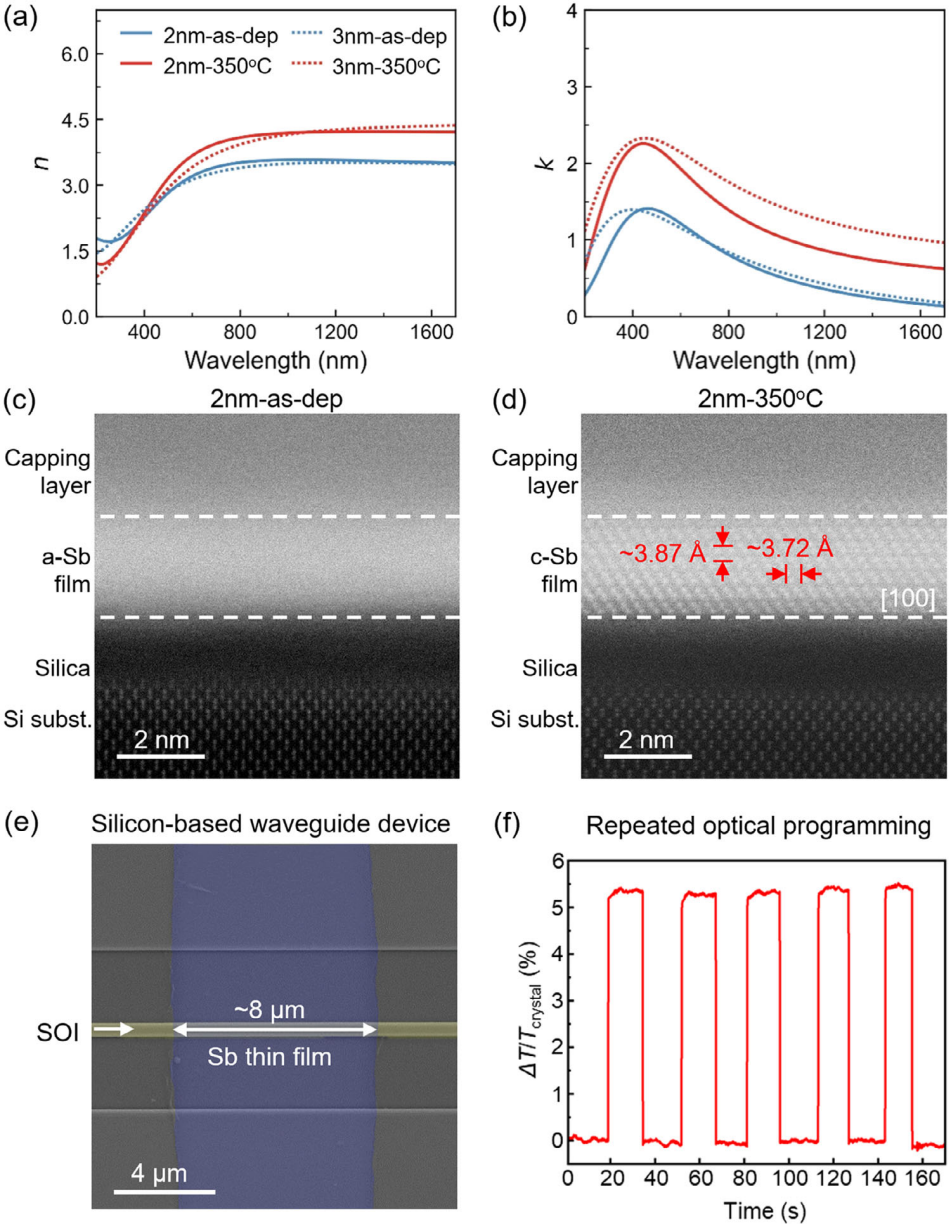
Phase‐change induced optical contrast in Sb thin films approaching the 2D limit. a,b) The refractive index *n* and extinction coefficient *k* of ≈2 and ≈3 nm Sb thin‐films measured by spectroscopic ellipsometry. c,d) The HAADF images of the as‐deposited and thermally annealed Sb thin films of ≈2 nm. e) SEM image of an SOI waveguide device (yellow false color) decorated with a 2‐nm‐thick Sb thin film of length ≈8 µm (cyan false color). f) Repeated all‐optical programming of the 2‐nm‐thick Sb waveguide device.

It is also noted that the overall electrical window shifted toward higher resistance, indicating reduced optical absorption due to the lower free‐carrier density. To assess the optical contrast, we carried out spectroscopic ellipsometry measurements on the as‐deposited a‐Sb thin films and on the 350 °C thermally annealed c‐Sb thin films. All Sb thin films were covered with a ZnS:SiO_2_ capping layer. The raw ellipsometry data and the fitting parameters can be found in Figure S6 and Table S1 (Supporting Information). As shown in **Figure** **6**a,b, the measured *n* and *k* values of the 3 nm Sb thin films showed a clear contrast window induced by crystallization. The contrast was consistently observed across the spectral range of 400–1700 nm, with average differences of ∆*n* ≈0.57 and ∆*k* ≈0.87. When the thickness of Sb was reduced to 2 nm, the contrast window in *n* remained essentially unchanged, while a moderately smaller contrast was observed in *k*, with ∆*k* ≈0.58. We note that different substrates and capping layers may influence the fitted *n* and *k* values. In the above experiments, we used a standard silicon substrate covered by a native SiO_2_ layer, which is commonly employed in PCM‐based optical platforms. The ZnS:SiO_2_ capping layer is transparent, non‐conductive, and widely used as a protective coating for PCM films. For comparison, we also prepared as‐deposited ≈2 and ≈3 nm Sb thin films on the same Si substrate but without a capping layer. As shown in Figure S7 (Supporting Information), the measured *n* and *k* values of the uncapped samples are comparable to those of the capped films (Figure 6a,b). However, these values are generally smaller than those reported by Cheng et al.,^[^ ^35^ ^]^ which may arise from differences in the ellipsometry setup or fitting procedures. Importantly, the trend of a reduced optical contrast window in thinner Sb films is consistent across our measurements and those of Cheng et al.

This experimentally observed thickness‐dependent optical trend was also consistent with our DFT calculations in Figure 2b. To make a direct comparison, we extracted the data of the 6 BL (2.1 nm) and 8 BL (2.8 nm) models and re‐plotted them in Figure S5 (Supporting Information), in which the 8BL model also showed a larger ∆*k* than the 6BL model. Therefore, we conclude that this optical trend with thin thickness is an intrinsic volume effect, which stems from the bonding differences in the crystalline phase and the amorphous phase with and without *p* orbital alignment. The sizable optical contrast of 2 nm Sb in the range of 400–1700 nm, makes it promising to develop non‐volatile color display and all‐optical photonic memory devices with enhanced state retention based on such 2D Sb.

To gain an in‐depth understanding of the atomic structures on the 2 nm Sb thin films, we performed cross‐sectional STEM experiments in the High Angle Annular Dark Field (HAADF) mode. The atomic‐scale images of the as‐deposited a‐Sb and thermally annealed c‐Sb are presented in Figure 6c,d, respectively. The former shows no visible lattice spots, and the latter shows clear Sb bilayer patterns with ≈6 intact bilayers with a [100] zone axis. The measured in‐plane and out‐of‐plane atomic spacings of 2 nm c‐Sb are 3.72 and 3.87 Å, as marked by the red arrows. Therefore, we fully demonstrated that the Sb thin film can still undergo phase transition at a very limited thickness of 2 nm, corresponding to only 12 atomic layers in the crystalline phase. We note that such a thickness limit in Sb is comparable to that of GeTe thin films obtained by the step‐by‐step growth via molecular beam epitaxy, in which 6 BL was also the bottom thickness limit to obtain crystalline GeTe.^[^ ^49^ ^]^ We note that crystallization of ultrathin Sb films (3–4 nm) with a well‐ordered out‐of‐plane orientation was also reported in Ref.,^[^ ^70^ ^]^ where no capping layer was used. In contrast, our AFM measurements show that amorphous Sb films of similar thickness (≈3 nm) undergo severe clustering after thermal annealing, forming a discontinuous crystalline film with very rough surface morphology (Figure S8, Supporting Information). The root‐mean‐square roughness increases from ≈0.159 nm in the as‐deposited amorphous film to ≈10.122 nm after annealing. A similar trend is observed for ≈2 nm Sb films. Therefore, in our case, a capping layer is required to avoid clustering. This discrepancy is probably due to differences in the deposition method and thermal processing: pulsed laser deposition at a substrate temperature of 210 °C in Ref.[70] vs magnetron sputtering followed by post‐annealing at 300 °C and above in our work, which can lead to distinct crystallization pathways in ultrathin Sb films.

To demonstrate that the 2 nm Sb thin film can still be reversibly switched optically, we fabricated silicon‐on‐insulator (SOI) waveguides and performed all‐optical pump–probe measurements. The back‐end‐of‐line (BEOL) fabrication was carried out via the multi‐project wafer (MPW) service of the Chongqing United Microelectronics Center Co., Ltd. (CUMEC), using a 180‐nm CMOS technology node. Prior to Sb deposition, reverse sputtering with argon plasma was applied, which is essential for ensuring clean waveguide surfaces. A ≈2 nm‐thick Sb film with a length of ≈8 µm was then deposited on the SOI waveguide (see Experimental Section for further details). Figure 6e shows a scanning electron microscopy (SEM) image of the fabricated device, where the silicon waveguide core and the Sb thin film are highlighted in yellow and cyan, respectively. Before optical testing, the device was annealed to obtain a fully crystalline state, ensuring improved programming consistency.

Next, we performed reversible all‐optical switching of the Sb waveguide devices using femtoseconds (fs) laser pulses.^[^ ^65^, ^68^, ^69^ ^]^ Specifically, we used 4 high‐power laser pulses and 200 weak laser pulses, each with a pulse width of 800 fs, to program the Sb waveguide device into an ON state with high transmittance (*T*_amor_) and an OFF state with low transmittance (*T*_cryst_), respectively. Figure 6f shows the measured transmittance response of the Sb waveguide device upon programming. A clear optical contrast Δ*T*/*T*_cry_ = ≈5.3% was observed, where Δ*T = T*_amor_−*T*_cryst_. These results confirm that ≈2 nm Sb can operate as a functional PCM in photonic waveguide memory devices.

We note that Aggarwal et al. reported reversible switching in ≈3 nm Sb waveguids,^[^ ^65^, ^68^, ^69^ ^]^ but their devices exhibited less stable transmittance over time: following amorphization pulses, the change in the optical readout initially reached ≈12%, but decreased to 2–6% within a few seconds. In contrast, the enhanced amorphous‐phase stability of ≈2 nm Sb leads to more robust and stable device performance. Nevertheless, the fabrication and optical characterization of Sb‐based devices at such reduced thickness remain challenging, and further efforts will be required to assess device variability in high‐density photonic arrays.

At last, we investigated the properties of closely related single‐element materials, namely tellurium^[^ ^71^ ^]^ and bismuth,^[^ ^72^ ^]^ to evaluate whether they could function as monatomic PCMs. Our DFT calculations yield ES values of 1.68 for crystalline Te and 1.41 for Bi. As shown in the ET─ES bonding map in Figure S9 (Supporting Information), Te falls within the covalent bonding regime, whereas Bi lies in the metavalent bonding region. These results already suggest that Te is unlikely to be a suitable PCM. Indeed, as reported by Zhu et al.,^[^ ^71^ ^]^ Te behaves as a monatomic phase‐change switch (PCS) material^[^ ^73^, ^74^, ^75^ ^]^ rather than a PCM. In their work, crystalline Te (with a sizable bandgap) served as the high‐resistance OFF state, whereas liquid Te (metallic) acted as the low‐resistance ON state. This represents a volatile phase transition, because the 20‐nm‐thick Te film crystallizes spontaneously after programming.^[^ ^71^ ^]^

In principle, it should be possible to obtain amorphous Te by reducing the film thickness to ≈2–3 nm, similar to the Sb case. We prepared ≈2 and ≈3 nm as‐deposited Te thin films and performed TEM and electrical measurements on them. As shown in Figure S10a (Supporting Information), the selected‐area electron diffraction (SAED) pattern of the ≈2 nm film displays a blurred halo ring, whereas sharp diffraction spots appear in the ≈3 nm film. This indicates that amorphous Te can be stabilized only when the film thickness is reduced to ≈2 nm. During in situ heating to 200 °C, the room‐temperature resistance initially decreases and then increases, which can be attributed to the fact that crystalline Te also tends to form a high‐resistance state. As a result, the achievable resistance contrast is very limited, meaning that elemental Te cannot be considered a PCM, even though an amorphous phase can be stabilized through confinement effects. Moreover, the resistance of the ≈2 nm Te films exceeds 10^10^ Ω, which is far too high for practical PCM applications.

According to the bonding calculation, Bi could in principle be a candidate for PCM applications. However, it is difficult to obtain amorphous Bi through confinement effects. The SAED patterns in Figure S10b show that both the ≈2 and ≈3 nm Bi thin films were already crystallized upon deposition. The resistance values barely changed after in situ heating. Hence, even a thickness of ≈2 nm is insufficient to stabilize as ‐deposited amorphous Bi thin film. In other words, Bi exhibits an even stronger tendency to crystallize than Sb. It may still be possible to obtain amorphous Bi in an ultrathin film of ≈1 nm, but such extreme scaling would make practical applications highly challenging. Overall, the unique balance between crystallization kinetics and property contrast makes Sb the only monatomic PCM successfully realized so far.

## 3 Conclusion

We have provided an atomistic understanding on the thickness‐dependent optical and bonding properties of monatomic PCM antimony by performing systematic ab initio simulations and FEM simulations. Reduction in electronic density of states near the Fermi level was found to contribute to the decrease of *k* in both crystalline and amorphous films. The weakening of MVB upon downscaling to a few atomic layers significantly impairs the optical excitation probability in the crystal, resulting in lower ∆*k* for thinner films. Nevertheless, the ∆*k* should be sufficiently large to determine the ON and OFF states for data encoding. Scaling the thickness of the Sb thin film down to 3 nm and below, capping layers became necessary in preventing the film rupture issue. With joint theoretical and experiment efforts, we demonstrated 2 nm to be the bottom thickness limit for functional Sb thin films, enabling a more robust amorphous stability with *T*_x_ ≈243 °C for nonvolatile electronic applications. Considering the sizable changes in electrical resistance, extinction coefficient in the near infrared range, and refractive indices in the visible light range, the 2 nm Sb thin film is suitable for nonvolatile photonic memory and reflective optical display applications. Our optical experiments demonstrate that the 2 nm Sb waveguide device can be reversibly programmed, exhibiting a sizable switching contrast of ≈5.3% Our combined multiscale simulations^[^ ^76^ ^]^ and experiments should serve as a typical example on how to optimize the thickness limit of materials for nonvolatile and reconfigurable optical applications from the atomic scale.

## 4 Experimental Section

### Materials Modeling

Ab initio molecular dynamics simulations for the amorphous models were performed using the second‐generation Car‐Parrinello scheme^[^ ^77^ ^]^ as implemented in the CP2K package,^[^ ^78^ ^]^ with an identical computational setup as in Ref.[38] The Perdew–Burke–Ernzerhof (PBE) functional^[^ ^79^ ^]^ and Goedecker pseudopotentials^[^ ^80^ ^]^ were used along with Grimme's D2 dispersion correction.^[^ ^81^ ^]^ Only the *Γ* point was used to sample the Brillouin zone, and the timestep was set to 2 fs. Structural relaxations and optical calculations were carried out using the Vienna Ab‐initio Simulation Package (VASP).^[^ ^82^ ^]^ The PBE functional and projector augmented‐wave (PAW) pseudopotentials^[^ ^83^ ^]^ were used with an energy cutoff of 500 eV, and the Grimme's D3 scheme^[^ ^84^ ^]^ was employed for dispersion corrections. Gaussian smearing with a width of 0.05 eV was used to describe partial occupancies of orbitals. For structural relaxation, the *k*‐point meshes were 13 × 13 × 5 and 13 × 13 × 1, respectively for crystalline bulk and slab models, and only the *Γ* point was used for the amorphous models. The convergence criteria were set as 10^−5^ eV for electronic self‐consistent loops and 0.01 eV Å^−1^ for ionic relaxation. For optical calculations, the *k*‐point density along each lattice direction was increased to at least twice for all the models for better convergence of the calculated optical properties, with a more stringent convergence threshold of 10^−6^ eV. The fully relaxed structures were then used for self‐consistent calculations using Quantum ESPRESSO,^[^ ^85^ ^]^ as the obtained electronic wavefunctions can be directly processed by the Critic2 code^[^ ^86^ ^]^ to compute the bonding indicators.^[^ ^66^, ^67^ ^]^ The QE calculations were applied with both norm‐conserving^[^ ^87^ ^]^ and PAW potentials together with PBE (the energy cutoff for wavefunctions and charge density was set as 80 and 320 Ry, respectively). In Critic2 calculations, the Bader's basins were determined based on wavefunctions, and then the Domain Overlap Matrices (DOM) were calculated to yield the delocalization (DIs) and localization indices (LIs), which measured the quantity of electron pairs shared between two basins and the number of electrons localized in a basin, respectively. The electrons shared (ES) between each pair of atoms was the twice of DI, and the electron transfer (ET) of an atom was determined by subtracting the number of electrons for the free reference atom from the number of localized electrons in its basin, and by dividing the resulting figure by the formal oxidation state.

### COMSOL Simulations

The RF module of COMSOL Multiphysics was used to perform finite element method simulations for waveguides working at the 1550 nm band. Frequency domain simulations were run after boundary mode analyses of input and output ports. The refractive indices of silica and silicon were described by the built‐in data provided by the software. A perfectly matched layer (PML) boundary condition was applied to truncate the simulation region in three dimensions, and the distance between PML and silicon waveguide is ≈2 µm. The meshes in each material region were constructed with the maximum mesh size being smaller than one‐fifth of the effective wavelength.

### Thin Film Preparation and Characterizations

The Sb thin films were deposited onto SiO_2_/Si substrate at room temperature via magnetron sputtering (AJA, Orion‐8), using a Sb target (99.99%) at a pressure of 4.7 mTorr with the radio frequency (RF) at ≈10 W in the high vacuum sputtering chamber. The thickness of Sb was controlled by the sputtering time, and the deposition rate was ≈0.72 nm min^−1^. A ZnS:SiO_2_ capping layer of ≈10 nm was deposited on top of Sb, except for the sample used in atomic force microscopy (AFM) experiments (using a SPM‐9700HT). Electrical measurements were performed using a Keithley 2636B source meter and an Instec mK200 hot stage instrument. The resistance of the films was measured in situ in an Ar atmosphere as a function of temperature using a two‐point probe method with a heating rate of 10 °C min^−1^, where the probe electrode was titanium. The Raman spectra were collected by using a Renishaw inVia Qontor Raman microscope with a solid‐state 532 nm laser for excitation. The laser power was set to 0.25 mW, and the exposure time was 1 s with 50 cycles. The spectroscopic ellipsometry measurements were performed with a UVISEL PLUS ellipsometer. The incidence angle was set to 70° with the light source of xenon lamps. The refractive indices of Sb were obtained by fitting the measured spectra using the CODE software (www.mtheiss.com) based on a multi‐layer model involving the substrate, the Sb thin film, and the capping layer. Two roughness layers were integrated into the established system and were positioned at the surface of the capping layer and at the interface between the capping layer and the Sb film, respectively. Both roughness layers were described using the Bruggeman effective medium approximation with a fixed volume fraction of 0.5. The dielectric model of amorphous Sb included a constant dielectric background and a Tauc–Lorentz oscillator, while an additional Drude contributor was incorporated for the crystalline phase. The dielectric functions of the substrate and the capping layer were determined independently based on reference samples (additional fitting details can be found in the caption of Table S1, Supporting Information). Such a method has been established in optical measurements of typical PCMs. The focused‐ion beams facility (FIB, Hitachi NX5000) was used to fabricate cross‐sectional TEM specimens. The high‐angle annular dark field scanning transmission electron microscopy (HAADF‐STEM) imaging experiments were performed on a JEM‐ARM200F microscope with double spherical aberration correctors. The SEM experiments were made using a Zeiss Sigma 300 scanning electron microscope.

### Waveguide Device Fabrication

The shallow‐etched SOI waveguides, featuring an etching depth of 150 nm and a width of 450 nm, were taped out via the MPW service provided by CUMEC. A selected region of the silicon dioxide upper cladding on the SOI waveguide was removed by reactive‐ion etching (RIE) to enable the subsequent deposition of the Sb thin film. Reverse sputtering using argon plasma etching was then carried out at a radio frequency power of 10 W for 5 min. Photolithography was performed to define the PCM deposition window using a negative‐tone photoresist (AZ 5214). A 2‐nm‐thick Sb thin film was deposited on the silicon waveguides via magnetron sputtering (AJA Orion‐8) with a sputtering power of 10 W, a working pressure of 4.7 mTorr, and a deposition rate of ≈0.72 nm min^−1^, calibrated from a prior Sb thin‐film deposition used for structural and optical characterizations. The Sb layer was subsequently capped with a ≈10‐nm‐thick indium–tin–oxide (ITO) layer. Finally, the photoresist was removed to lift off the Sb and ITO thin films outside the patterned windows, leaving patch‐shaped Sb/ITO stacks integrated on the silicon waveguides.

## Conflict of Interest

The authors declare no conflict of interest.

## Supporting information



Supporting Information

## Data Availability

Data supporting this work isavailable at https://caid.xjtu.edu.cn/info/1003/2071.htm.

## References

[1] Z. Wang , H. Wu , G. W. Burr , C. S. Hwang , K. L. Wang , Q. Xia , J. J. Yang , Nat. Rev. Mater. 2020, 5, 173.

[2] A. Sebastian , M. L. Gallo , R. Khaddam‐Aljameh , E. Eleftheriou , Nat. Nanotechnol. 2020, 15, 529. 32231270 10.1038/s41565-020-0655-z

[3] W. Zhang , R. Mazzarello , M. Wuttig , E. Ma , Nat. Rev. Mater. 2019, 4, 150.

[4] N. Youngblood , C. A. Ríos Ocampo , W. H. P. Pernice , H. Bhaskaran , Nat. Photon. 2023, 17, 561.

[5] B. J. Shastri , A. N. Tait , T. Ferreira De Lima , W. H. P. Pernice , H. Bhaskaran , C. D. Wright , P. R. Prucnal , Nat. Photon. 2021, 15, 102.

[6] M. Wuttig , N. Yamada , Nat. Mater. 2007, 6, 824. 17972937 10.1038/nmat2009

[7] M. Wuttig , H. Bhaskaran , T. Taubner , Nat. Photon. 2017, 11, 465.

[8] M. Xu , X. Mai , J. Lin , W. Zhang , Y. Li , Y. He , H. Tong , X. Hou , P. Zhou , X. Miao , Adv. Funct. Mater. 2020, 30, 2003419.

[9] W. Zhang , E. Ma , Mater. Today 2020, 41, 156.

[10] G. Wang , J. Shen , Y. He , Z. Han , W. Huang , H. Wang , Z. Cheng , P. Zhou , Adv. Mater. 2025, 37, 2419444. 10.1002/adma.202419444 40059565

[11] Q. Xu , W. Zhang , X.‐B. Li , C. Wang , M. Xu , S. Tang , S. Yuan , M. Xu , Z. Wang , X. Miao , M. Wuttig , InfoMat 2025, 7, 70006.

[12] N. Yamada , E. Ohno , K. Nishiuchi , N. Akahira , M. Takao , J. Appl. Phys. 1991, 69, 2849.

[13] N. Yamada , T. Matsunaga , J. Appl. Phys. 2000, 88, 7020.

[14] T.‐T. Jiang , X.‐D. Wang , J.‐J. Wang , H.‐Y. Zhang , L. Lu , C. Jia , M. Wuttig , R. Mazzarello , W. Zhang , E. Ma , Fundam. Res. 2024, 4, 1235. 39431143 10.1016/j.fmre.2022.09.010 PMC11489497

[15] H. Iwasaki , M. Harigaya , O. Nonoyama , Y. Kageyama , M. Takahashi , K. Yamada , H. Deguchi , Y. Ide , Jpn. J. Appl. Phys. 1993, 32, 5241.

[16] T. Matsunaga , J. Akola , S. Kohara , T. Honma , K. Kobayashi , E. Ikenaga , R. O. Jones , N. Yamada , M. Takata , R. Kojima , Nat. Mater. 2011, 10, 129. 21217690 10.1038/nmat2931

[17] W. Zhang , I. Ronneberger , P. Zalden , M. Xu , M. Salinga , M. Wuttig , R. Mazzarello , Sci. Rep. 2014, 4, 6529. 25284316 10.1038/srep06529 PMC4185410

[18] F. Rao , K. Ding , Y. Zhou , Y. Zheng , M. Xia , S. Lv , Z. Song , S. Feng , I. Ronneberger , R. Mazzarello , W. Zhang , E. Ma , Science 2017, 358, 1423. 29123020 10.1126/science.aao3212

[19] J. Akola , R. O. Jones , Science 2017, 358, 1386. 29242333 10.1126/science.aaq0476

[20] J. Huang , B. Chen , G. Sha , H. Gong , T. Song , K. Ding , F. Rao , Nano Lett. 2023, 23, 2362. 36861962 10.1021/acs.nanolett.3c00262

[21] S. Hu , B. Liu , Z. Li , J. Zhou , Z. Sun , Comput. Mater. Sci. 2019, 165, 51.

[22] X.‐P. Wang , X.‐B. Li , N.‐K. Chen , J. Bang , R. Nelson , C. Ertural , R. Dronskowski , H.‐B. Sun , S. Zhang , npj Comput. Mater. 2020, 6, 31.

[23] Y.‐T. Liu , X.‐B. Li , H. Zheng , N.‐K. Chen , X.‐P. Wang , X.‐L. Zhang , H.‐B. Sun , S. Zhang , Adv. Funct. Mater. 2021, 31, 2009803.

[24] Y. Xu , X. Wang , W. Zhang , L. Schafer , J. Reindl , F. Vom Bruch , Y. Zhou , V. Evang , J. J. Wang , V. L. Deringer , E. Ma , M. Wuttig , R. Mazzarello , Adv. Mater. 2021, 33, 2006221. 33491816 10.1002/adma.202006221 PMC11468882

[25] S. Sun , X. Wang , Y. Jiang , Y. Lei , S. Zhang , S. Kumar , J. Zhang , E. Ma , R. Mazzarello , J.‐J. Wang , W. Zhang , npj Comput. Mater. 2024, 10, 189.

[26] Y. Xie , W. Kim , Y. Kim , S. Kim , J. Gonsalves , M. BrightSky , C. Lam , Y. Zhu , J. J. Cha , Adv. Mater. 2018, 30, 1705587. 10.1002/adma.201705587 29327386

[27] C. Kim , D. Kang , T.‐Y. Lee , K. H. P. Kim , Y.‐S. Kang , J. Lee , S.‐W. Nam , K.‐B. Kim , Y. Khang , Appl. Phys. Lett. 2009, 94, 193504.

[28] M. Salinga , B. Kersting , I. Ronneberger , V. P. Jonnalagadda , X. T. Vu , M. L. Gallo , I. Giannopoulos , O. Cojocaru‐Miredin , R. Mazzarello , A. Sebastian , Nat. Mater. 2018, 17, 681. 29915424 10.1038/s41563-018-0110-9

[29] J. Shen , W. Song , K. Ren , Z. Song , P. Zhou , M. Zhu , Adv. Mater. 2023, 35, 2208065. 10.1002/adma.202208065 36719053

[30] J. J. Hauser , Phys. Rev. B 1974, 9, 2623.

[31] B. Chen , X.‐P. Wang , F. Jiao , L. Ning , J. Huang , J. Xie , S. Zhang , X.‐B. Li , F. Rao , Adv. Sci. 2023, 10, 2301043. 10.1002/advs.202301043 PMC10477879 37377084

[32] W. Zhang , E. Ma , Nat. Mater. 2018, 17, 654. 29915426 10.1038/s41563-018-0114-5

[33] D. T. Yimam , B. J. Kooi , ACS Appl. Mater. Interfaces 2022, 14, 13593. 35266381 10.1021/acsami.1c23974 PMC8949766

[34] D. Dragoni , J. Behler , M. Bernasconi , Nanoscale 2021, 13, 16146. 34542138 10.1039/d1nr03432d

[35] Z. Cheng , T. Milne , P. Salter , J. S. Kim , S. Humphrey , M. Booth , H. Bhaskaran , Sci. Adv. 2021, 7, abd7097. 10.1126/sciadv.abd7097 PMC7775754 33523855

[36] S. Aggarwal , T. Milne , N. Farmakidis , J. Feldmann , X. Li , Y. Shu , Z. Cheng , M. Salinga , W. H. Pernice , H. Bhaskaran , Nano Lett. 2022, 22, 3532. 35451845 10.1021/acs.nanolett.1c04286 PMC9101065

[37] S. Aggarwal , N. Farmakidis , B. Dong , J. S. Lee , M. Wang , Z. Xu , H. Bhaskaran , Nanophotonics 2024, 13, 2223. 39634499 10.1515/nanoph-2023-0654 PMC11501610

[38] X. Shen , Y. Zhou , H. Zhang , V. L. Deringer , R. Mazzarello , W. Zhang , Nanoscale 2023, 15, 15259. 37674458 10.1039/d3nr03536k

[39] M. Shi , J. Li , M. Tao , X. Zhang , J. Liu , Mater. Sci. Semicond. Process. 2021, 136, 106146.

[40] C. Ríos , M. Stegmaier , P. Hosseini , D. Wang , T. Scherer , C. D. Wright , H. Bhaskaran , W. H. P. Pernice , Nat. Photon. 2015, 9, 725.

[41] Z. Fang , R. Chen , J. Zheng , A. I. Khan , K. M. Neilson , S. J. Geiger , D. M. Callahan , M. G. Moebius , A. Saxena , M. E. Chen , C. Rios , J. Hu , E. Pop , A. Majumdar , Nat. Nanotechnol. 2022, 17, 842. 35788188 10.1038/s41565-022-01153-w

[42] D. Wang , L. Zhao , S. Yu , X. Shen , J.‐J. Wang , C. Hu , W. Zhou , W. Zhang , Mater. Today 2023, 68, 334.

[43] W. Zhou , X. Shen , X. Yang , J. Wang , W. Zhang , Int. J. Extreme Manuf. 2024, 6, 022001.

[44] J. Xie , J. Yan , H. Han , Y. Zhao , M. Luo , J. Li , H. Guo , M. Qiao , Nano‐Micro Lett. 2025, 17, 179. 10.1007/s40820-025-01693-5 PMC11896963 40067576

[45] S. Zhang , Z. Yan , Y. Li , Z. Chen , H. Zeng , Angew. Chem. Int. Ed. 2015, 54, 3112. 10.1002/anie.201411246 25564773

[46] J. Ji , X. Song , J. Liu , Z. Yan , C. Huo , S. Zhang , M. Su , L. Liao , W. Wang , Z. Ni , Y. Hao , H. Zeng , Nat. Commun. 2016, 7, 13352. 27845327 10.1038/ncomms13352 PMC5116078

[47] X. Wang , J. Song , J. Qu , Angew. Chem. Int. Ed. 2019, 58, 1574. 10.1002/anie.201808302 30137673

[48] P. Hosseini , C. D. Wright , H. Bhaskaran , Nature 2014, 511, 206. 25008527 10.1038/nature13487

[49] R. Wang , W. Zhang , J. Momand , I. Ronneberger , J. E. Boschker , R. Mazzarello , B. J. Kooi , H. Riechert , M. Wuttig , R. Calarco , NPG Asia Mater. 2017, 9, 396.

[50] I. Ronneberger , Z. Zanolli , M. Wuttig , R. Mazzarello , Adv. Mater. 2020, 32, 2001033. 10.1002/adma.202001033 32537877

[51] P. Kerres , Y. Zhou , H. Vaishnav , M. Raghuwanshi , J. Wang , M. Haser , M. Pohlmann , Y. Cheng , C. F. Schon , T. Jansen , C. Bellin , D. E. Burgler , A. R. Jalil , C. Ringkamp , H. Kowalczyk , C. M. Schneider , A. Shukla , M. Wuttig , Small 2022, 18, 2201753. 10.1002/smll.202201753 35491494

[52] S. Caravati , M. Bernasconi , M. Parrinello , J. Phys.: Condens. Matter 2010, 22, 315801. 21399368 10.1088/0953-8984/22/31/315801

[53] W. Welnic , S. Botti , L. Reining , M. Wuttig , Phys. Rev. Lett. 2007, 98, 236403. 17677924 10.1103/PhysRevLett.98.236403

[54] H. Zhang , X. Wang , W. Zhang , Opt. Mater. Express 2022, 12, 2497.

[55] X.‐D. Wang , W. Zhou , H. Zhang , S. Ahmed , T. Huang , R. Mazzarello , E. Ma , W. Zhang , npj Comput. Mater. 2023, 9, 136.

[56] W. Zhang , H. Zhang , S. Sun , X. Wang , Z. Lu , Wang, J.‐J. W , C. Jia , C.‐F. Schön , R. Mazzarello , E. Ma , M. Wuttig , Adv. Sci. 2023, 10, 2300901. 10.1002/advs.202300901 PMC10214272 36995041

[57] V. Wang , N. Xu , J.‐C. Liu , G. Tang , W.‐T. Geng , Comput. Phys. Commun. 2021, 267, 108033.

[58] K. Shportko , S. Kremers , M. Woda , D. Lencer , J. Robertson , M. Wuttig , Nat. Mater. 2008, 7, 653. 18622406 10.1038/nmat2226

[59] M. Wuttig , V. L. Deringer , X. Gonze , C. Bichara , J. Y. Raty , Adv. Mater. 2018, 30, 1803777. 10.1002/adma.201803777 30318844

[60] M. Zhu , O. Cojocaru‐Miredin , A. M. Mio , J. Keutgen , M. Kupers , Y. Yu , J. Y. Cho , R. Dronskowski , M. Wuttig , Adv. Mater. 2018, 30, 1706735. 10.1002/adma.201706735 29572962

[61] J. Y. Raty , M. Schumacher , P. Golub , V. L. Deringer , C. Gatti , M. Wuttig , Adv. Mater. 2019, 31, 1806280. 10.1002/adma.201806280 30474156

[62] Y. Cheng , S. Wahl , M. Wuttig , Phys. Status Solidi RRL 2020, 15, 2000482.

[63] B. J. Kooi , M. Wuttig , Adv. Mater. 2020, 32, 1908302. 10.1002/adma.201908302 32243014

[64] O. Cojocaru‐Mirédin , Y. Yu , J. Köttgen , T. Ghosh , C. F. Schön , S. Han , C. Zhou , M. Zhu , M. Wuttig , Adv. Mater. 2024, 36, 2403046. 39520347 10.1002/adma.202403046 PMC11636162

[65] L. Guarneri , S. Jakobs , A. Hoegen , S. Maier , M. Xu , M. Zhu , S. Wahl , C. Teichrib , Y. Zhou , O. Cojocaru‐Mirédin , M. Raghuwanshi , C.‐F. Schön , M. Drögeler , C. Stampfer , R. P. S. M. Lobo , A. Piarristeguy , A. Pradel , J.‐Y. Raty , M. Wuttig , Adv. Mater. 2021, 33, 2102356. 34355435 10.1002/adma.202102356 PMC11468997

[66] J.‐Y. Raty , C. Gatti , C.‐F. Schön , M. Wuttig , Phys. Status Solidi RRL 2021, 15, 2000534.

[67] J.‐Y. Raty , C. Bichara , C.‐F. Schön , C. Gatti , M. Wuttig , Proc. Natl. Acad. Sci. U.S.A. 2024, 121, e2316498121. 38170754 10.1073/pnas.2316498121 PMC10786265

[68] D. Lencer , M. Salinga , B. Grabowski , T. Hickel , J. Neugebauer , M. Wuttig , Nat. Mater. 2008, 7, 972. 19011618 10.1038/nmat2330

[69] J.‐Y. Raty , M. Wuttig , J. Phys. D: Appl. Phys. 2020, 53, 234002.

[70] D. T. Yimam , M. Ahmadi , B. J. Kooi , Mater. Today Nano 2023, 23, 100365.

[71] J. Shen , S. Jia , N. Shi , Q. Ge , T. Gotoh , S. Lv , Q. Liu , R. Dronskowski , S. R. Elliott , Z. Song , M. Zhu , Science 2021, 374, 1390. 34882462 10.1126/science.abi6332

[72] F. Hoff , P. Kerres , T. Veslin , A. R. Jalil , T. Schmidt , S. Ritarossi , J. Kottgen , L. Bothe , J. Frank , C. F. Schon , Y. Xu , D. Kim , J. Mertens , J. Mayer , R. Mazzarello , M. Wuttig , Adv. Mater. 2025, 37, 2416938. 39740119 10.1002/adma.202416938 PMC11837888

[73] X.‐D. Wang , W. Zhang , E. Ma , Sci. Bull. 2022, 67, 888. 10.1016/j.scib.2022.01.020 36546018

[74] Z. Zhao , M. Zhang , Q. Yang , T. Gotoh , Q. Ge , N. Shi , Y. Sun , J. Zhao , Y. Sui , R. Jiang , H. Yu , S. R. Elliott , Z. Song , M. Zhu , Adv. Funct. Mater. 2025, 35, 2423940.

[75] Y. Sun , B. Li , T. Yang , Q. Yang , H. Yu , T. Gotoh , C. Shi , J. Shen , P. Zhou , S. R. Elliott , H. Li , Z. Song , M. Zhu , Adv. Funct. Mater. 2025, 35, 2408725.

[76] X. Shen , R. Chu , Y. Jiang , W. Zhang , Acta Metall. Sin. 2024, 60, 1362.

[77] T. D. Kühne , M. Krack , F. R. Mohamed , M. Parrinello , Phys. Rev. Lett. 2007, 98, 066401. 17358962 10.1103/PhysRevLett.98.066401

[78] J. Hutter , M. Iannuzzi , F. Schiffmann , J. VandeVondele , WIREs Comput. Mol. Sci. 2014, 4, 15.

[79] J. P. Perdew , K. Burke , M. Ernzerhof , Phys. Rev. Lett. 1996, 77, 3865. 10062328 10.1103/PhysRevLett.77.3865

[80] S. Goedecker , M. Teter , J. Hutter , Phys. Rev. B 1996, 54, 1703. 10.1103/physrevb.54.1703 9986014

[81] S. Grimme , J. Comput. Chem. 2006, 27, 1787. 16955487 10.1002/jcc.20495

[82] G. Kresse , J. Hafner , Phys. Rev. B 1993, 47, 558. 10.1103/physrevb.47.558 10004490

[83] G. Kresse , D. Joubert , Phys. Rev. B 1999, 59, 1758.

[84] S. Grimme , J. Antony , S. Ehrlich , H. Krieg , J. Chem. Phys. 2010, 132, 154104. 20423165 10.1063/1.3382344

[85] P. Giannozzi , S. Baroni , N. Bonini , M. Calandra , R. Car , C. Cavazzoni , D. Ceresoli , G. L. Chiarotti , M. Cococcioni , I. Dabo , A. Dal Corso , S. de Gironcoli , S. Fabris , G. Fratesi , R. Gebauer , U. Gerstmann , C. Gougoussis , A. Kokalj , M. Lazzeri , L. Martin‐Samos , N. Marzari , F. Mauri , R. Mazzarello , S. Paolini , A. Pasquarello , L. Paulatto , C. Sbraccia , S. Scandolo , G. Sclauzero , A. P. Seitsonen , et al., J. Phys.: Condens. Matter. 2009, 21, 395502. 21832390 10.1088/0953-8984/21/39/395502

[86] A. Otero‐de‐la‐Roza , E. R. Johnson , V. Luaña , Comput. Phys. Commun. 2014, 185, 1007.

[87] D. R. Hamann , Phys. Rev. B 2013, 88, 085117.

