# Supplementary material for: Atomistic Understanding of 2D Monatomic Phase‐Change Material for Non‐Volatile Optical Applications
Source: Adv Sci (Weinh). 2026 Feb 11;13(16):e13157. doi: 10.1002/advs.202513157 (PMC13042991; doi:10.1002/advs.202513157)
Supplement: Supplementary file 1 — Supporting Information [file ADVS-13-e13157-s001.docx]

**Supporting Information**

Atomistic understanding of two-dimensional monatomic phase-change material for non-volatile optical applications

*Hanyi Zhang^#^, Xueqi Xing^#^, Jiang-Jing Wang*, Chao Nie, Yuxin Du, Junying Zhang, Xueyang Shen, Wen Zhou*, Matthias Wuttig, Riccardo Mazzarello*, Wei Zhang**


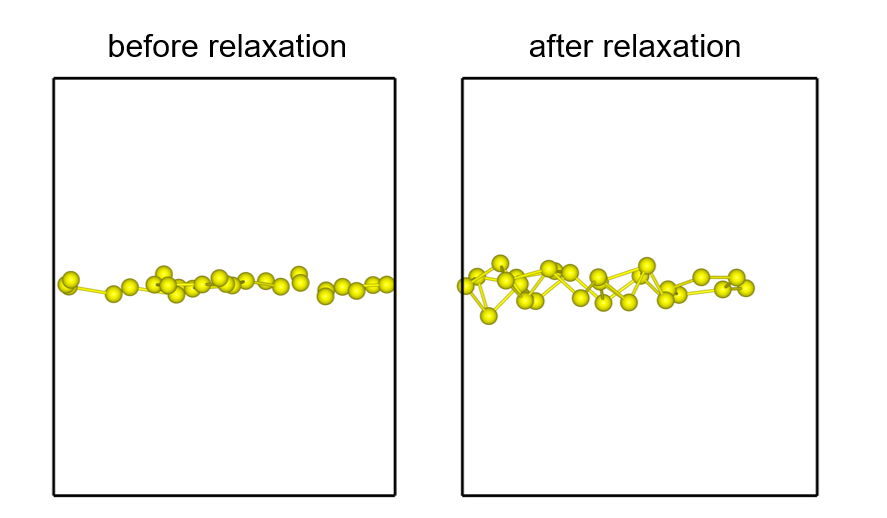


**Figure S1.** The atomic coordinates of the thinnest a-Sb model before and after relaxation. The initial coordinates were taken from bulk a-Sb model along the vertical direction over ~0.2 nm. The limited thickness leads to clustering of atoms and a rupture of the slab.


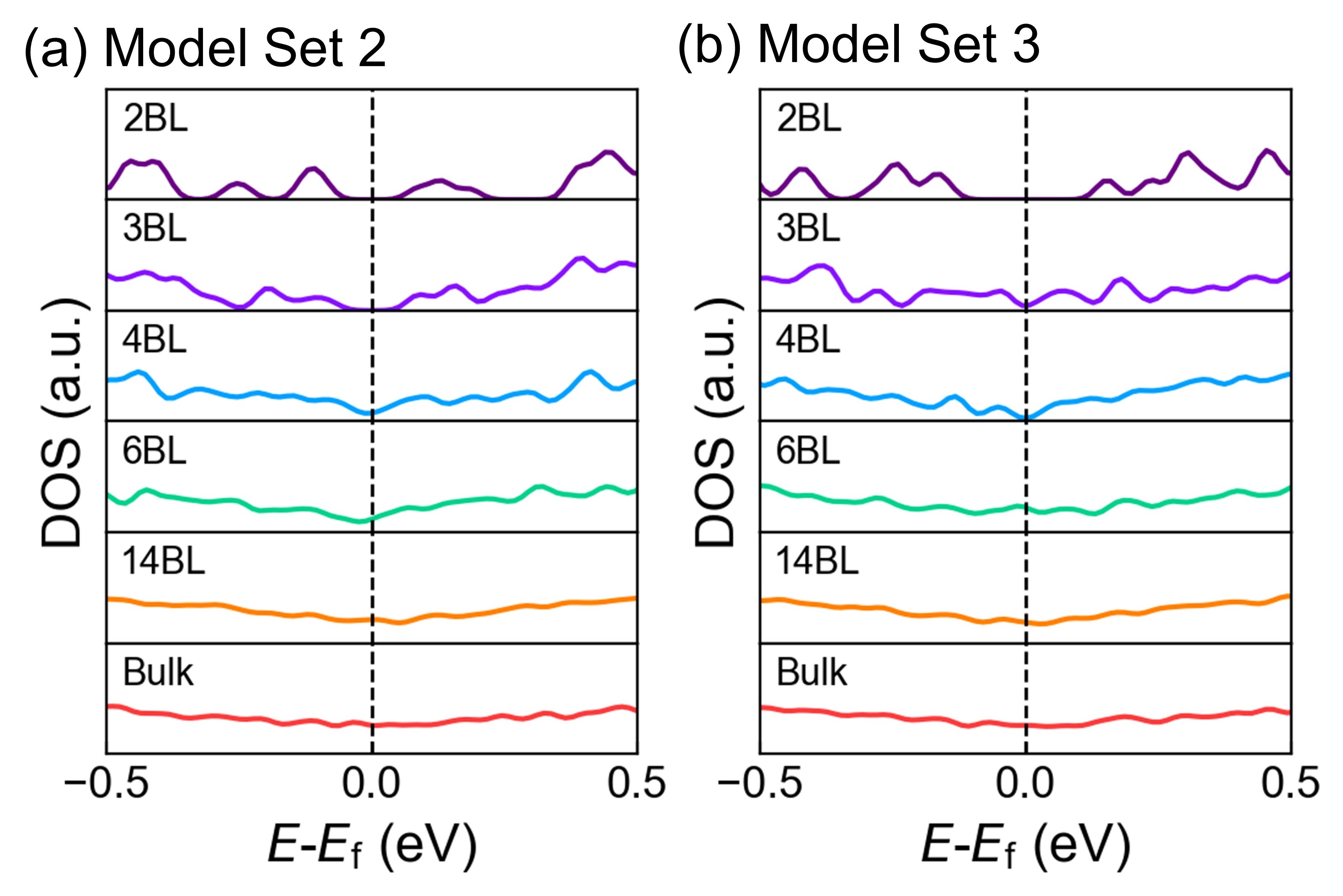


**Figure S2.** The electronic density of states (DOS) for (a) the second and (b) the third sets of amorphous models.


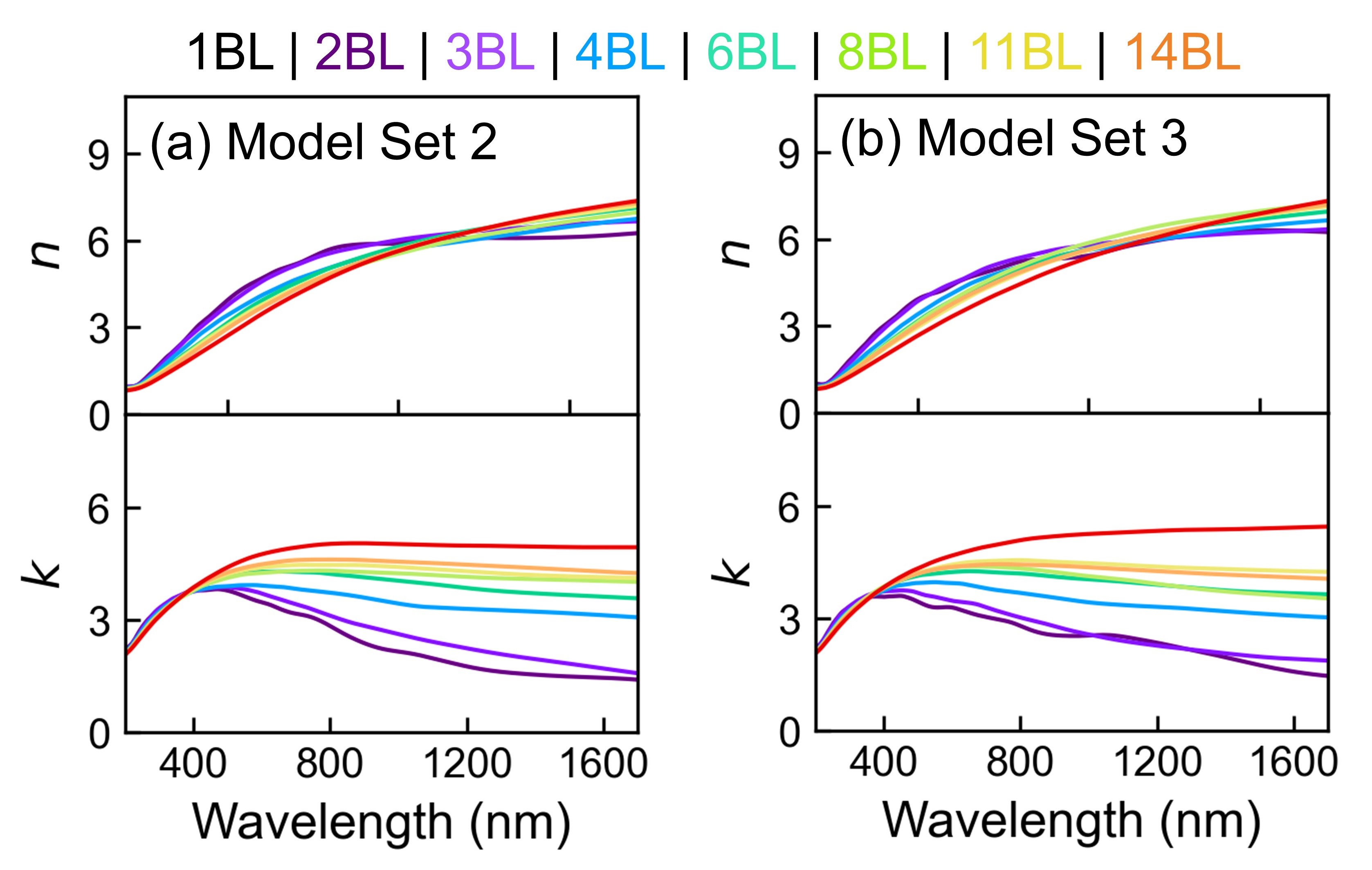


**Figure S3.** The refractive index (*n*) and extinction coefficient (*k*) for (a) the second and (b) the third sets of amorphous models.


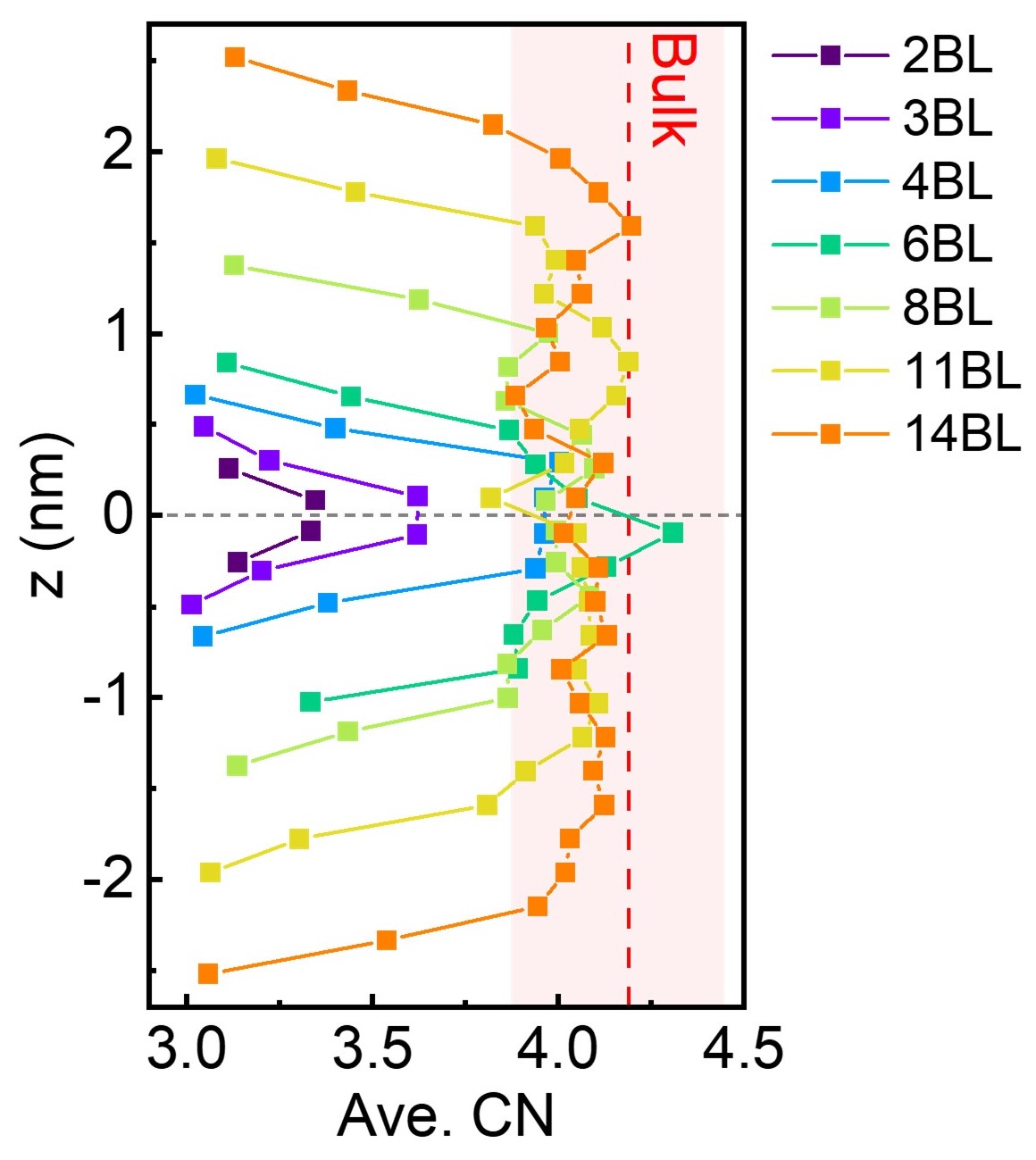


**Figure S4.** Average coordination number for each layer in amorphous models. The interatomic cutoff is set as 3.4 Å. The red shaded area shows the fluctuation range of coordination number in bulk amorphous models.


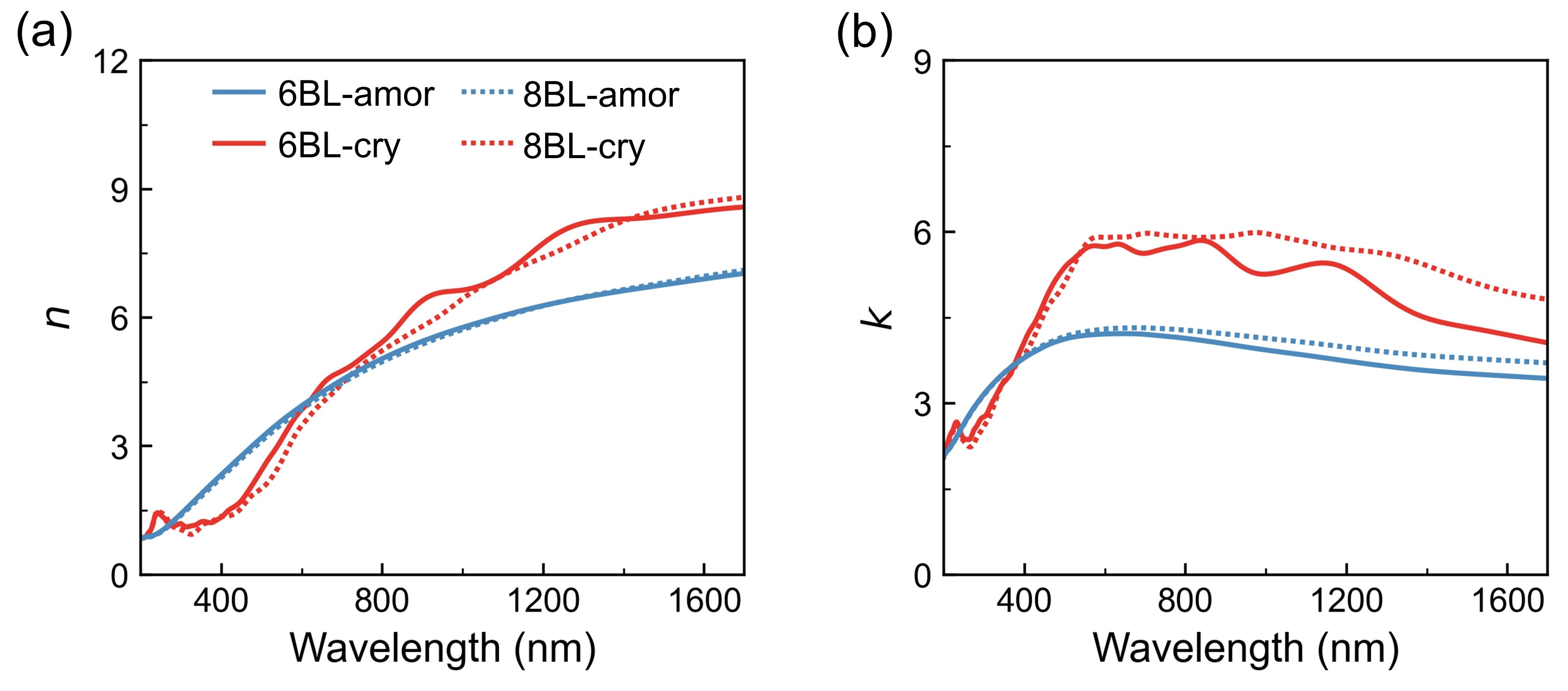


**Figure S5.** The DFT-calculated refractive index *n* and extinction coefficient *k* of the 6BL (2.1 nm) and 8BL (2.8 nm) Sb models, as extracted from Fig. 2b. Note that these calculations were performed using the PBE functional, which is known to underestimate the size of the energy gap, leading to stronger optical responses. Nevertheless, the thickness-dependent optical trend was consistently observed in both DFT calculations and ellipsometry experiments.


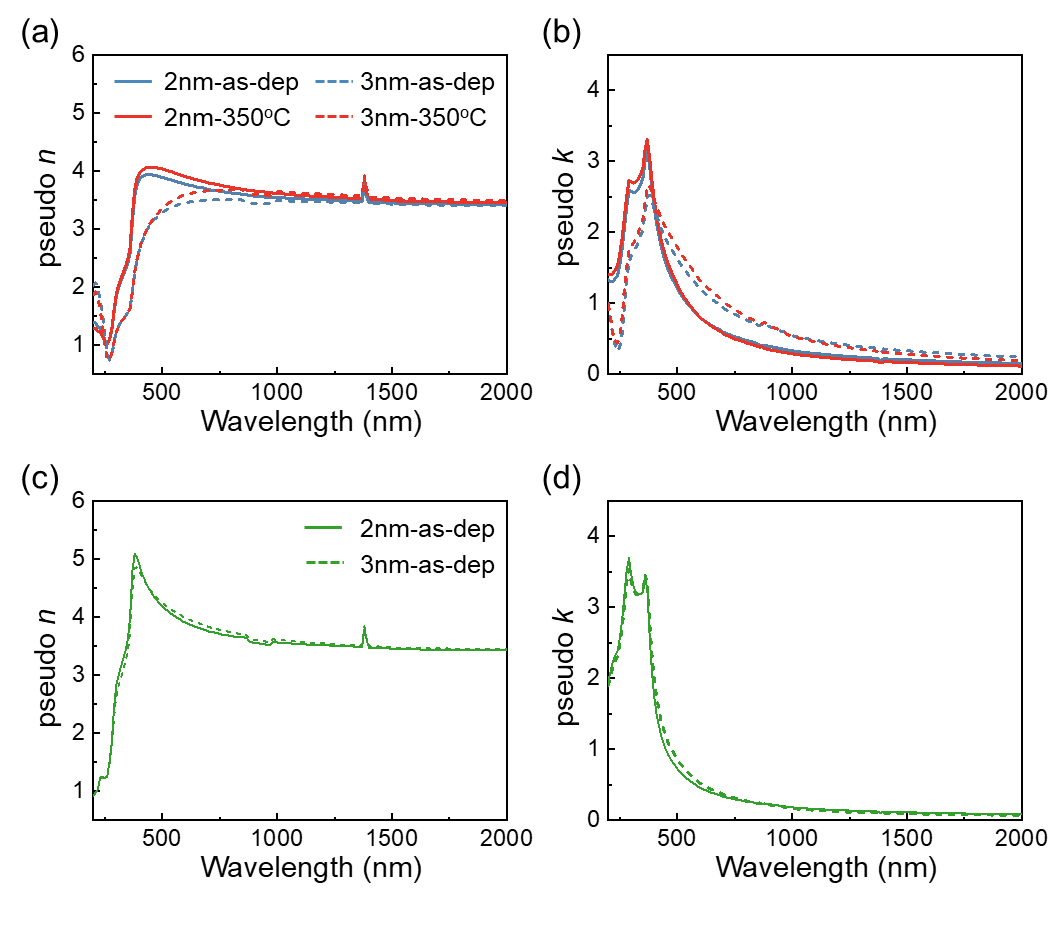


**Figure S6.** Raw ellipsometry data, namely (a) pseudo *n* and (b) pseudo *k*, corresponding to the *n* and *k* data fitting of the Sb thin films shown in Fig. 6a and 6b.

|  | 2nm-as-dep | 2nm-350^o^C | 3nm-as-dep | 3nm-350^o^C |
| --- | --- | --- | --- | --- |
| $\omega_{0}$ (eV) | 2.54 | 2.61 | 3.02 | 2.64 |
| $A$ (eV) | 33.21 | 43.44 | 46.02 | 50.61 |
| $\omega_{\tau}$ (eV) | 3.04 | 2.98 | 5.00 | 3.84 |
| $\omega_{\text{Gap}}$ (eV) | 0.40 | 0.13 | 0.39 | 0.06 |
| $\varepsilon_{\text{const}}$ | 4.57 | 3.69 | 3.29 | 2.35 |
| $\Omega_{P}$ (eV) | - | 1.68 | - | 1.96 |
| $\Omega_{\tau}$ (eV) | - | 2.42 | - | 2.40 |
| Sb thickness (nm) | 2.00 | 1.86 | 3.19 | 2.80 |
| Sb/capping roughness layer thickness (nm) | 0.30 | 0.10 | 0.28 | 0.42 |
| Capping thickness (nm) | 4.98 | 4.80 | 10.00 | 9.50 |
| Capping surface thickness (nm) | 0.10 | 0.11 | 0.11 | 0.16 |

·

**Table S1.** Final fitting parameters obtained from the CODE calculations for the ellipsometry analysis of 2 nm and 3 nm Sb thin films with capping layers. The dielectric model of amorphous Sb consisted of a constant background term, $\varepsilon_{\text{const}}$, and a Tauc–Lorentz oscillator. The Tauc–Lorentz model expresses the imaginary part of the susceptibility as:

$$\chi\left( \omega\right)=\frac{1}{\omega}\frac{A\omega_{0}\omega_{\tau}(\omega-\omega_{\text{Gap}})^{2}}{(\omega^{2}-\omega_{0}^{2})^{2}+\omega^{2}\omega_{\tau}^{2}}\Theta\left( \omega-\omega_{\text{Gap}} \right),$$

where $A$, $\omega_{\tau}$, $\omega_{0}$ and $\omega_{\text{Gap}}$ are fitting parameters representing the amplitude, damping constant, resonance frequency and optical bandgap, respectively. An additional Drude term was incorporated to describe the free-carrier contribution in the crystalline phase:

$\chi\left( \tilde{\nu} \right)=-\frac{\Omega_{p}^{2}}{\tilde{\nu}^{2}+i\tilde{\nu}\Omega_{\tau}},$

where $\Omega_{P}$ and $\Omega_{\tau}$ are fitting parameters corresponding to the plasma frequency and damping constant, respectively.


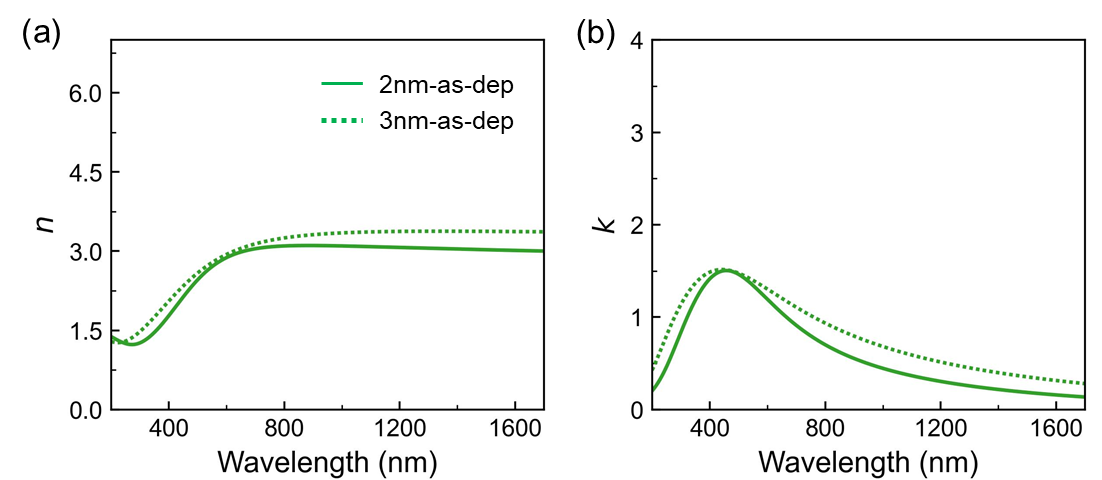


**Figure S7.** Measured refractive index *n* and extinction coefficient *k* of as-deposited ~2 nm and ~3 nm Sb films without capping layers.


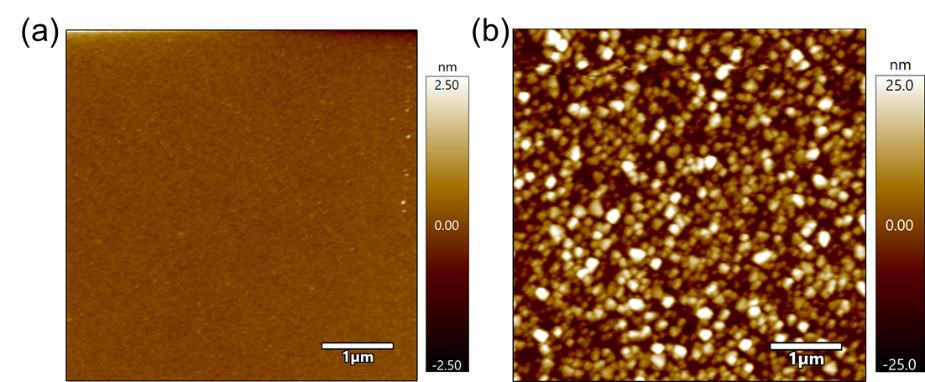


**Figure S8.** AFM images of ~3 nm Sb thin films without a capping layer. The two sets of samples were prepared in a single sputtering run at room temperature. (a) AFM image of the first as-deposited amorphous Sb thin film, showing a smooth surface with a root-mean-square roughness (*R*_q_) of ~0.159 nm. (b) AFM image of the second Sb sample after annealing at 300 °C. The measured *R*_q_ value of ~10.122 nm is much larger than that of the initial as-deposited amorphous sample.


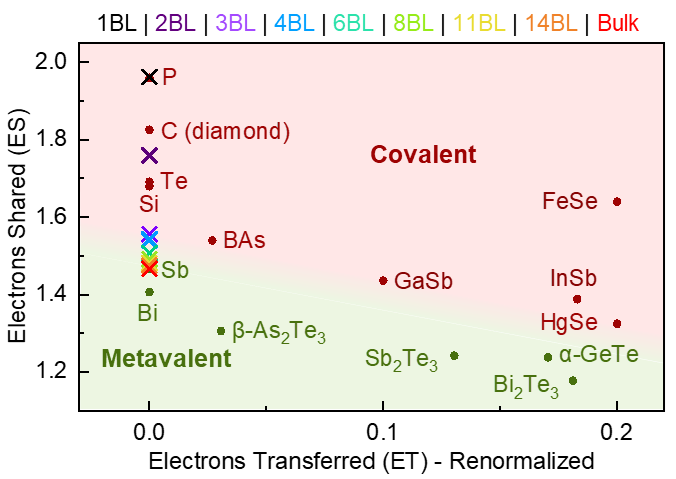


**Figure S9.** Location of the crystalline Te and Bi models on the bonding map defined by the Electrons Transferred (ET) and Electrons Shared (ES) indicators.


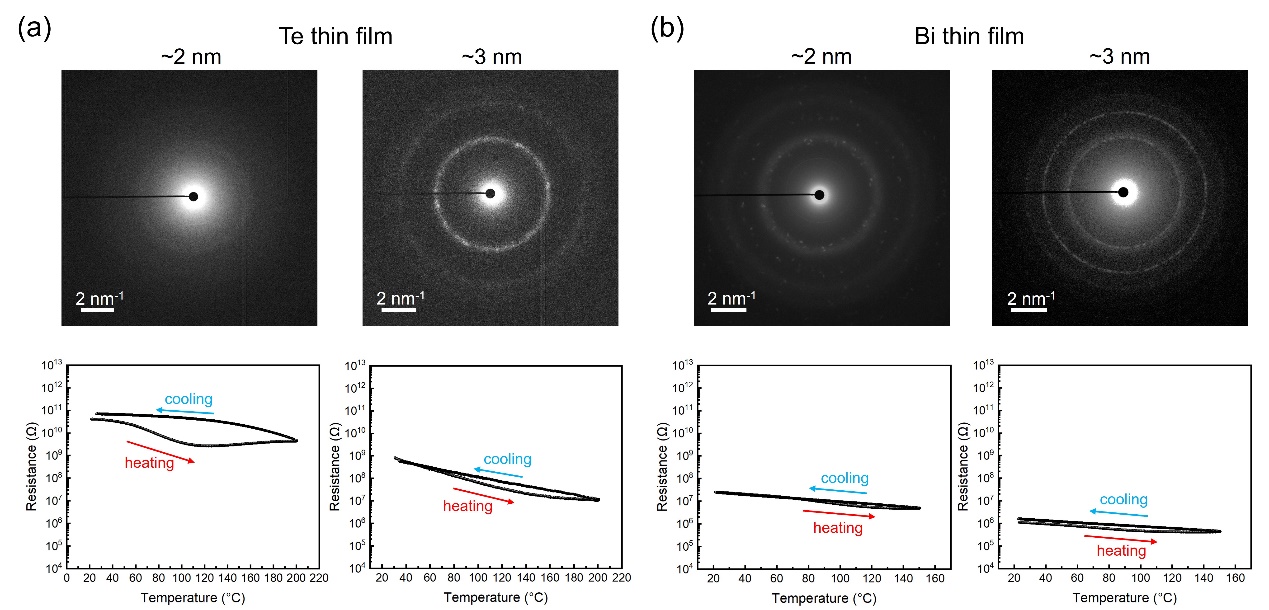


**Figure S10.** Selected area electron diffraction (SAED) patterns and resistance–temperature measurements of as-deposited (a) Te and (b) Bi thin films with thicknesses of ~2 nm and ~3 nm. The Te and Bi thin films were deposited onto the same silicon substrate at room temperature via magnetron sputtering, using high-purity Te (99.99%) and Bi (99.99%) targets. The depositions were performed in a high-vacuum sputtering chamber at a working pressure of 4.7 mTorr with an RF power of ~10 W. A ~10-nm ZnS:SiO₂ capping layer was then deposited on top of the thin films.
